# Inhibition of PDGFRβ alleviates endothelial cell apoptotic injury caused by DRP-1 overexpression and mitochondria fusion failure after mitophagy

**DOI:** 10.1038/s41419-023-06272-3

**Published:** 2023-11-18

**Authors:** Xiaohong An, Xiao Ma, Heng Liu, Jing Song, Tiange Wei, Rongzhan Zhang, Xiao Zhan, Hongyang Li, Jia Zhou

**Affiliations:** 1 School of Traditional Chinese Pharmacy, China Pharmaceutical University, Nanjing, 211198 China; 2 Yunnan Characteristic Plant Extraction Laboratory, Yunnan Yunke Characteristic Plant Extraction Laboratory Co., Ltd, Kunming, 650106 China; 3 Yunnan Provincial Key Laboratory of Entomological Biopharmaceutical R&D, Dali University, Dali, 671000 China; 4 Laboratory Animal Center, Xiamen University, Xiamen, 361102 China; 5 Institute of Dermatology, Chinese Academy of Medical Sciences and Peking Union Medical College, Nanjing, 210042 China

**Keywords:** Receptor pharmacology, Cardiovascular diseases

## Abstract

**Abstract:**

Kawasaki disease (KD), described as *“mucocutaneous lymph node syndrome”*, affects infants and toddlers. Patients with KD suffer from an inflammatory cascade leading to vasculitis with a predilection for coronary arteries. While the symptoms and pathogenesis of KD have received more and more attention, the precise mechanisms are still debated. Researches show that endothelial dysfunction process in KD leads to arterial damage and affect clinical outcome. In this study, we constructed a Candida albicans water soluble fraction (CAWS)-induced KD murine model and penetrated investigating the mechanisms behind endothelial dysfunction. CAWS-induced mice presented remarkably elevated vascular endothelial cell growth factor (VEGF) levels. Abundant expression of VEGF was documented in all vessels that showed edema from acute KD. It has been reported that Platelet-derived growth factor (PDGF) co-expression normalizes VEGF-induced aberrant angiogenesis. Hyperexpression of PDGFRβ was induced in the thickened medial layer and vascular endothelium of KD mice. Masitinib (Mas) is an oral tyrosine kinase inhibitor of numerous targets, which can selectively target PDGFR signaling. We set out to explore whether Mas could regulate coronary pathology in KD. Mas administration significantly reduced the VEGF-induced endothelial cells migration. NOX4 was activated in vascular endothelial cells to produce more ROS. Mitochondrial dysregulated fission and mitophagy caused by DRP-1 overexpression precipitated the arterial endothelial cells injury. Here, mitophagy seemed to work as the driving force of DRP-1/Bak/BNIP3-dependent endothelial cells apoptosis. In summary, how mitophagy is regulated by DRP-1 under pathologic status is critical and complex, which may contribute to the development of specific therapeutic interventions in cardiovascular diseases patients, for example Masatinib, the inhibitor of PDGFRβ.

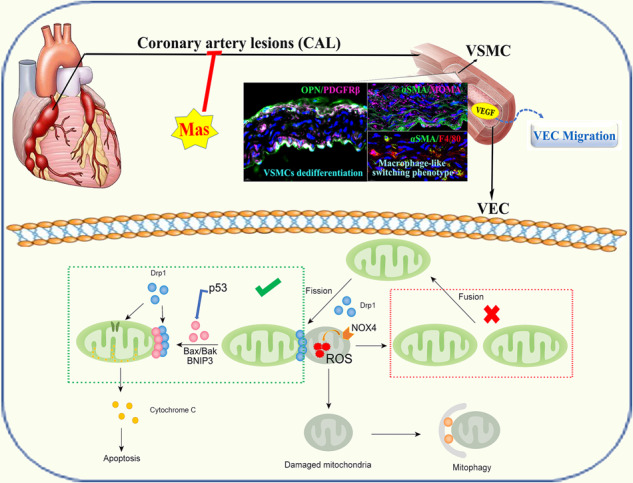

**Facts and questions:**

- Kawasaki disease causing systemic vasculitis, affects infants and toddlers.
- Coronary artery injury remains the major causes of morbidity and mortality.
- DRP-1 overexpression induces DRP-1/Bak/BNIP3-dependent endothelial cells apoptosis.
- PDGFRβ was high-expressed in the thickened medial layer of CAWS-induced KD mice.
- Inhibition of PDGFRβ signaling alleviates arterial endothelial cells injury.

## Introduction

Kawasaki disease (KD), which was described as “*mucocutaneous lymph node syndrome*”, affects infants and toddlers, and causes systemic vasculitis [1, 2]. When initially described, it was considered as a benign disease with self-limiting course [3, 4] and the potential for coronary artery complications of KD was not appreciated. Autopsy studies later revealed the relevance between coronary artery complications and KD [2]. Over time, it has now been realized that KD may cause several other cardiac complications as well [5]. Coronary artery aneurysms may develop nearly one-quarter of all cases, to make matters worse, their development is clinically silent in most cases and may be recognized only years later at the time of sudden death or myocardial infarction [6, 7]. Coronary and peripheral artery injury remain the major causes of morbidity and mortality, despite current medical and surgical options.

While the symptoms and pathogenesis of KD have received more and more attention, the precise mechanisms are still debated. Activation of the immune system is a central feature of KD, and concentrations of many pro-inflammatory cytokines and chemokines, including tumor necrosis factor α (TNF-α), interleukins (IL) 1, 6, and 18, are higher than normal during the acute phase of the disease [2]. The leading hypothesis is a prevalent infectious trigger producing a clinically significant inflammatory disease within a genetically susceptible host [8]. Whatever the inciting factor, patients with KD suffer from an inflammatory cascade leading to vasculitis with a predilection for coronary arteries. Coronary dilatation and aneurysms then develop over weeks. This inflammatory insult produces endothelial dysfunction and increased vascular permeability. Endothelial dysfunction promotes vasoconstriction and increases susceptibility to thrombogenesis by accelerated platelet aggregation, hypercoagulability, and hypofibrinolysis. Beyond that, endothelial dysfunction is closely related to the risk factors for atherosclerosis, it is necessary to longitudinally evaluate systemic endothelial function in KD patients.

Activate monocytes/macrophages seem to have an important role in KD, which have been found in the vessel walls of patients who died and in skin biopsy samples from patients in the acute phase of the disease [9–11]. Skin biopsy samples in KD demonstrates gap formation and fenestration of endothelial cells, inducing perivascular edematous changes [12]. Vascular endothelial cell growth factor (VEGF) is the most potent angiogenic factor produced by endothelial cells, fibroblasts, T cells, and macrophages, plays an important role in exacerbating angiogenesis and inflammation [13]. It induces normal or aberrant angiogenesis depending on its dose in the microenvironment around each producing cell in vivo. As one candidate, high concentrations of VEGF increased vascular permeability, which could be the cause of the edema in the vessel wall [2, 12]. Abundant expression of VEGF was documented immunohistochemically in all vessels that showed edema from acute KD [14]. Another cytokine, platelet-derived growth factor (PDGF), which plays a critical role in blood vessel formation during embryonic life [15, 16]. PDGF is a major mitogen for many cell types of mesenchymal origin and is regulated by effectors in pathogenic pathways, such as thrombin, TNF-α, IL-6, and high glucose [17]. It has been reported that PDGF co-expression normalizes VEGF-induced aberrant angiogenesis [18]. The switch from normal to aberrant angiogenesis does not depend exclusively on VEGF dose, but rather on the balance between endothelial stimulation by VEGF and pericyte recruitment by PDGF, such that PDGF codelivery ensures normal and functional microvascular growth despite high or uncontrolled VEGF expression. VEGF promotes endothelial cell growth, while PDGF stabilizes blood vessels by recruiting pericytes [19].

PDGF acts on cells by binding to homo- or heterodimers of the two PDGF receptor (PDGFR) proteins, PDGFRα and PDGFRβ. Binding of ligand to PDGFRβ induces its dimerization and autophosphorylation, which initiate a number of signal transduction pathways [20]. PDGFRβ is expressed in mesenchyme, particularly in vascular smooth muscle cells (VSMCs) and pericytes. PDGFRβ and its downstream pathways play prominent role in multiple cellular functions, including proliferation, migration, matrix deposition, and immediate early gene induction [21]. In inflammatory microenvironment, PDGF /PDGFRβ and the downstream Src-Akt signal pathway control mesenchymal stem cells migration [22]. Human natural killer cells express high levels of PDGFRβ to elevate survival following IL-15 stimulation [23]. It has also been reported that PDGFRβ participated in KD vascular injury. Functional miR-223, which was delivered by hyperactive platelets isolated from patients with KD, promoted VSMC differentiation via downregulation of PDGFRβ [24]. Platelet activation is thought to be involved in the acute phase of KD to develop vascular inflammation in a vicious circle manner as follows: cells activated by platelet-derived microparticles produce inflammatory cytokines to induce monocytes, macrophages, neutrophils, and vascular endothelial cells to express tissue factors, which promote the generation of thrombin to mediate further activation of platelets [25–27]. Endothelial damage from KD vasculitis could promote platelet activation and direct contact between platelets and VSMC [27].

Endothelial dysfunction process in KD leads to arterial damage and affect clinical outcome, needing to be well studied. Though PDGFRβ has not typically been associated with endothelial cells susceptibility, PDGF/PDGFRβ axis is a critical modulator of the angiogenic response evoked in endothelial cells [28]. How PDGFRβ and its downstream pathways participate in endothelial dysfunction has not been elaborated up to now. Masitinib (Mas), an oral tyrosine kinase inhibitor of PDGFR, has been gaining attention as a potential therapeutic agent in the clinical setting, due to its effects on inflammatory pathogenesis [29]. Considering the involvement of inflammation in KD injuries and the potential therapeutic effect of Mas, we investigated the effect of Mas on abnormal endothelial dysfunction during KD vasculitis.

## Materials and method

### Mice

Male C57BL/J mice weighing 18–22 g, SPF grade, provided by the Model Animal Research Institute of Xiamen University, meet the quality standards of common laboratory animals. License number: SCXK (Min) 2018-0003. The mice were housed in SPF grade independent ventilation cages (IVC), with free access to water and food. The temperature of the rearing room was 21–24 °C and the relative humidity was 40%–70%. All procedures and assessments were approved by the Animal Ethics Committee of the School of Chinese Materia Medica, China Pharmaceutical University. These experiments were carried out in accordance with the National Institutes of Health Guide for the Care and use of Laboratory Animals (National Institutes of Health Publication No. 80-23, revised in 1996). Before performing the experiments, all animals were randomized into experimental groups, and the indices were measured by operators blinded to the study procedures.

### Preparation of CAWS

Candida albicans strain CMCC(F)98001 was purchased from the Beijing Sanyao Technology Co., Ltd, stored at 25 °C on Sabauroud’s agar (Difco, USA), and passaged once every 3 months. Candida albicans water soluble fraction (CAWS) was prepared from C. albicans strain CMCC(F)98001 in accordance with conventional methods. The procedure used was as follows: 5 L of medium (C-limiting medium) was added to a glass incubator and cultured for 2 days at 27 °C with air supplied at a rate of 5 L/min and rotation at 400 rpm. Following the culture, an equal volume of ethanol was added and after the mixture was allowed to stand overnight, the precipitate was collected. The precipitate was dissolved in 250 mL of distilled water, ethanol was added and the mixture was left to stand overnight. The precipitate was collected and dried with acetone to obtain CAWS.

### Administration schedule for induction of KD and Mas treatments

CAWS (4 mg/mouse) was administered intraperitoneally for five consecutive days to each mouse in week 1. Then, mice were treated with masitinib at 75 mg/kg/day or vehicle (the saline) from the 14th day for 14 consecutive days. The hearts of the animals were fixed with 10% neutral formalin and prepared in paraffin blocks. Tissue sections were stained with HE stains. Cells were prepared from the spleen and cultured.

### Enzyme-linked immunosorbent assay (ELISA)

The amount of interleukin (IL)-1β, 6 and 18, factor-alpha (TNF-α) in murine plasma were determined by (Elabscience, E-EL-M0037, E-EL-M0044, E-EL-M0730, E-EL-M3063), respectively. Briefly, add 100 μL each dilution of standard, blank, and sample into the appropriate wells and incubate for 90 min at 37 °C. Decant the liquid from each well, immediately add 100 μL of Biotinylated Detection Ab working solution to each well. Cover the plate with a new sealer and incubate for 1 h at 37 °C. Decant the solution from each well, add 350 μL of wash buffer to each well. Soak for 1 min and aspirate or decant the solution from each well and pat it dry against clean absorbent paper. Repeat this wash step 3 times. Add 100 μL of HRP Conjugate working solution to each well. Cover the plate with a new sealer. Incubate for 30 min at 37 °C. Add 90 μL of Substrate Reagent to each well. Cover the plate with a new sealer. Incubate for about 15 min at 37 °C. Protect the plate from light. Add 50 μL of Stop Solution to each well. Note: adding the stop solution should be done in the same order as the substrate solution. Determine the optical density (OD value) of each well at once with a micro-plate reader set to 450 nm.

### Immunohistochemical staining and immunofluorescence

The aorta tissues were fixed in 4% paraformaldehyde and embedded with paraffin. For histopathologic examination, all fixed organs were processed for embedding in paraffin, applied 4-µm sections, and stained with hematoxylin and eosin (H&E). For tissue immunohistochemical and immunofluorescence, 4-µm of paraffin sections were freshly prepared and dried in 45 °C calorstat. Sections were dewaxed with fresh dimethylbenzene twice for 5–10 min at a time. Sections were the postfixed in absolute ethyl alcohol for 5 min, 85% ethyl alcohol for 5 min, 75% ethyl alcohol for 5 min, and distilled water for 5 min. After incubation with protease K that contained no DNase for 15–30 min at 20–37 °C, sections were washed 3 times with PBS. After the sections were slightly dried, drew a circle around the tissue with a histochemical pen, added a self-fluorescence quenching agent into the circle for 5 min, and rinsed with water for 10 min. Added BSA in the circle and incubated for 30 min. Sections were incubated with primary antibodies overnight, then second antibodies warm incubated for 50 min. The sections were placed in PBS (pH 7.4) and washed three times for 5 min each time. After the slices were slightly dried, sections were counterstained with DAPI for identification of cell nuclei. The sections were placed in PBS (pH 7.4) and washed three times for 5 min each time. After the slices were slightly dried, sealed with anti-fluorescence quencher, and then stored in a 4-degree dark section box. Images were obtained by using 80i Fluorescence Microscope (Nikon). Primary antibodies used were PDGFRβ (Abcam, ab203491), α-SMA (BOSTER, BM0002), OPN (PTG, 22952-1-AP), MOMA (Abcam, ab33451), F4/80 (CST,70076), eNOS (Abcam, ab76198), CD31 (Abcam, ab182981), iNOS (Bioss, BS-0162R), VEGF (Abclonal, A12303), RhoA (Affinity, df5046), ROCK1 (PTG, 21850-1-AP), NOX4 (Abclonal, A22149), DRP-1 (PTG,12957-1-ap), HIF-1α (BOSTER, BM4083), BNIP3 (Abcam, ab109362), LC3B (PTG, 18725-1-AP), Parkin (Bioss, BS-23687), p62 (BOSTER, BA2849), LAMP1 (PTG, 67300-1-IG), TOMM20 (PTG, 11802-1-AP), Mfn1/2 (Abclonal, A12771), Bak (CST, 12105), cytochrome c (CST, 4280 T), E2F3 (Bioss, bs-21438r), p53 (PTG, 21891-1-AP).

### Western blot analysis

For western blotting, cell pellets were lysed in modified Cell lysis buffer (Beyotime, P0013) containing 1 mM PMSF (Beyotime, ST506). Protein concentration in the lysates was determined using Enhanced BCA Protein Assay Kit (Beyotime, P0010), and 40 μg of protein per well was separated by SDS-PAGE and transferred to a PDVF membrane. Primary antibodies used were RhoA (CST, 2117 S), ROCK1 (CST, 4035) (ab134181), MFF (CST, 84580), p-DRP-1(Ser616) (CST, 4494), DRP-1 (CST, 8570), BNIP3/NIX (CST, 12396), PINK1 (Abcam, ab23707), Parkin (Abcam, ab159224), Beclin 1 (CST, 3495), Atg12 (CST, 4180), Atg101(CST, 13492), LC3I/II (CST, 12741), p62 (CST, 39749), LAMP1 (Abcam, ab24179), OPA1 (CST, 80471), cytochrome c (CST, 4280 T), cleaved caspase 3 (CST, 9661 S), Bax (CST, 2772 S), GAPDH (CST, 60004), β-Tubulin (Proteintech, 66240). Proteins were visualized by using an ECL detection system. Densitometric analysis was performed by using the Tanon 4600SF (Tanon Science & Technology, Shanghai, China), to scan signals. Western blot assay results reported here are representative of at least 3 experiments.

### Cell culture

Human Coronary Artery Endothelial cells (HCAEC) (Catalog #6020) and human umbilical vein endothelial cell (HUVEC) (Catalog #8000) were purchased from Sciencell. Cells were cultured in a complete medium, composed of Endothelial Cell Medium (ECM) (Sciencell, Catalog #1001), Endothelial Cell Growth Supplement (ECGS, Sciencell, Cat #1052), 5% fetal bovine serum (FBS, Sciencell, Catalog #0025), 1% (v/v) penicillin/streptomycin, HEPES and bicarbonate buffered to maintain pH 7.4, at 37 °C with 5% CO_2_/95% air.

### Scratch wound assay

Serum-starved HUVEC cells were cultured to attain a 90% confluence in six-well plates and subsequently damaged using the tip of a pipette. For prevention of cell proliferation, the cells were reacted with mitomycin C (10 µg/mL) for 1 h, followed by incubation with indicated concentrations of Mas (0.3, 1, 3 µM). After a 6-h/12-h incubation, the migrated area was captured and measured using a phase-contrast microscope. Serum-starved HUVEC cells were incubated with VEGF (1 nM, Peprotech), VEGF (1 nM) + Mas (1 µM) or not for 12 h, the migrated area was captured and measured using a phase-contrast microscope.

### Transwell migration assay

A transwell migration assay was conducted using the 24-well Millicell Hanging Cell Culture Inserts of 8.0-µm pore size (Millipore, Switzerland). The serum-starved HCAEC were incubated in serum-free ECM supplemented with Mas (0.3,1, 3 µM) or not, and then they were seeded into each of the upper sections of the chamber plates for 12 h, allowing cellular invasiveness through the membrane. The cells from the upper side of the membrane were scraped off with cotton swabs, and invaded cells were fixed in 90% methanol and stained with crystal violet (0.1%). The relative invasive cells were photographic recorded under a microscope across six randomly selected areas at 100× magnification per each well.

The serum-starved HCAEC were incubated in serum-free ECM supplemented with VEGF (1 nM), VEGF (1 nM) + Mas (1 µM) or not, and then they were seeded into each of the upper sections of the chamber plates for 12 h, allowing cellular invasiveness through the membrane. The relative invasive cells were photographic recorded under a microscope across six randomly selected areas at 100× magnification per each well.

### Transmission electron microscopy

After discarding the cultured cell medium without rinsing, HUVEC cells were fixed in 2.5% (vol/vol) glutaraldehyde and collected in the centrifuge tube. After discarding the fixative, add new electron microscope fixative for 2 h at room temperature, and then transfer to 4° for preservation. The samples were imaged using a JEM-1400Flash TEM (JEOL). For electron microscopy quantification, 5 image fields were selected for each sample.

### Statistics

We used 12 mice/group in the in vivo experiments, while 6 for histopathological section and others for molecular biology experiments. Data were analyzed by *t*-test, one- and two-way ANOVA, followed by post hoc tests as appropriate, using GraphPad Prism software (GraphPad Software Inc.). *P* value < 0.05 was considered statistically significant.

## Results

### Inducement of cardiovascular dysfunction and hyperexpression of PDGFRβ in KD murine model by CAWS

**Fig. 1:**
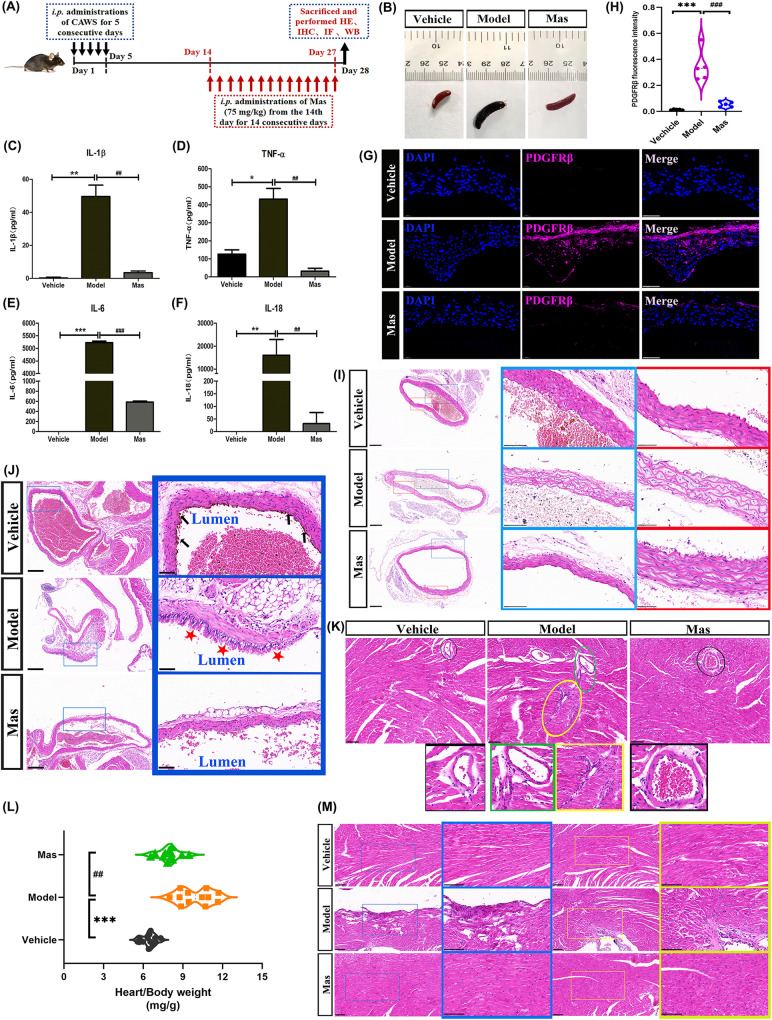
Inducement of cardiovascular dysfunction and hyperexpression of PDGFRβ in KD murine model by CAWS. **A** Schematic approach of experimental procedure. **B** Typical photographs of spleen. **C**–**F** Serum levels of IL-1β, IL-6, IL-18, and TNF-α were examined by Elisa. Every three serum samples of each group are blended into one tube for Elisa examination (*n* = 12 per group, mixed to formed 4 test samples). **G** Representative immunofluorescence images for detecting PDGFRβ (magenta) expressions within aorta, DAPI (blue) (*n* = 5 per group). Scale bar: 50 μm. **H** PDGFRβ fluorescence intensity. **I** H&E staining showing the morphological characteristics of mouse aorta (*n* = 4 per group). Enlarged images of area of interesting (AOI) were indicated with blue and red block diagrams. Scale bar: 200 μm (low magnification), 100 μm (high magnification in blue block diagrams), and 50 μm (high magnification in red block diagrams). **J** H&E staining showing the morphological characteristics of main coronary artery (*n* = 4 per group). Enlarged images of area of interesting (AOI) were indicated with blue block diagrams. Scale bar: 200 μm (low magnification), 50 μm (high magnification). **K** H&E staining showing the morphological characteristics of coronary bifurcation arteritis (*n* = 4 per group). Enlarged images of area of interesting (AOI) were indicated with oval frames. Scale bar: 100 μm (low magnification). **L** Heart weight/body weight ratio of each group. Each data point represents a biological replicate. **M** H&E staining showing the morphological characteristics of myocardium (*n* = 4 per group). Enlarged images of area of interesting (AOI) were indicated with blue and yellow block diagrams. Scale bar: 100 μm (low magnification) and 50 μm (high magnification). One-way ANOVA was followed by *post hoc* Tukey’s test. All results are presented as mean ± SEM. *P < 0.05, **P < 0.01, ***P < *0.001 vs* vehicle group, ^*##*^*P* < *0.01*, ^#*##*^*P* < *0.001 vs* model group.

The schematic approach of experimental procedure was showed in Fig. 1A. The typical pathological changes of KD vasculitis were successfully induced in mice. Compared with the vehicle group, the spleen of the model group was enlarged and black in color (Fig. 1B). We examined the serum levels of pro-inflammatory cytokines. In comparison to vehicle mice, CAWS-induced mice presented remarkably elevated IL-1β, IL-6, IL-18 and TNF-α levels (Fig. 1C–F). Nevertheless, Mas administration significantly reduced the release of above inflammatory cytokines in CAWS-triggered murine models of KD. Hence, the CAWS-induced murine model of KD presented the activation of pro-inflammatory cytokines.

Considering the vital role of PDGF in pathogenesis of vascular damage in KD, in this experiment, the expression of its receptor, PDGFRβ was examined. Hyperexpression of PDGFRβ was induced in the thickened medial layer and vascular endothelium of CAWS-induced KD mice (Fig. 1G, H). For vehicle mice, the aorta intima was smooth and intact, and the endothelial cells were neatly arranged. In the CAWS-induced model group, the vessel wall was partially thickened, the intima had obvious edema, thickening, and degeneration, the vascular endothelial cells (VECs) were arranged disorderly, and small vacuoles were found in the cytoplasm, surrounded by inflammatory cell infiltration. In the Mas administration group, the thickness of the vessel wall was basically restored, the endothelial thickening and edema were significantly reduced, and it was basically restored to smoothness, and the endothelial cells were densely arranged. This aortic injury could be significantly reduced by the administration of Mas (Fig. 1I).

There is a strong predilection for the coronary arteries in KD. In the acute phase of KD, the coronary artery is infiltrated by numerous active inflammatory cells, which results in the destruction of elastic tissue of the vascular media with subsequent formation of artery aneurysms [30, 31]. Attempting to induce changes mimicking the arteritis of KD, we employed a well-established murine model of KD in which intraperitoneal injection of CAWS induces vascular inflammation [32]. Consistent with pathology reported in the previous literature, our CAWS-induced KD mice developed progressive abdominal main coronary artery and its bifurcation arteritis. All blood vessels are composed of two distinct cell types: endothelial cells and mural cells. Whereas endothelial cells form the inner vessel wall, the mural cells associate with and coat the endothelial cell tube. In vehicle mice, the undulated elastic fibers are clearly visible. The black arrow represents endothelial cells and the inner elastic membrane. And there also be accumulation of inflammatory cell infiltration under the intima (as indicated by the red pentagram) (Fig. 1J). A large number of inflammatory cells infiltrate around the smaller coronary branches (as indicated by the green and yellow oval) (Fig. 1K). In the Mas treatment group, the vascular injury was reversed in some extent, though a few inflammatory cells could still be seen.

Moreover, CAWS-induced mice had significantly elevated heart weight/body weight ratio compared with vehicle mice, but the ratio was markedly alleviated by Mas treatment (Fig. 1L). In the vehicle group, the cardiomyocytes of the heart tissue were closely arranged, with normal morphology and without obvious lesions. In the CAWS-induced model group, the heart tissue was seriously damaged, some myocardial cells were coagulated and necrotic, the cytoplasm was stained with eosin, and there was focal inflammatory cell infiltration in local areas. In the Mas treatment group, the structure and morphology of the heart were basically normal, and the myocardial cells were arranged in a roughly orderly manner (Fig. 1M). Nevertheless, the cardiac function and dimension were remarkably maintained following Mas administration. Above data demonstrated the CAWS-induced cardiac and vascular inflammation, Mas could reverse these damages.

### VSMCs dedifferentiated to macrophage-like switching phenotype and activation of pro-inflammatory cytokines in KD murine model

**Fig. 2:**
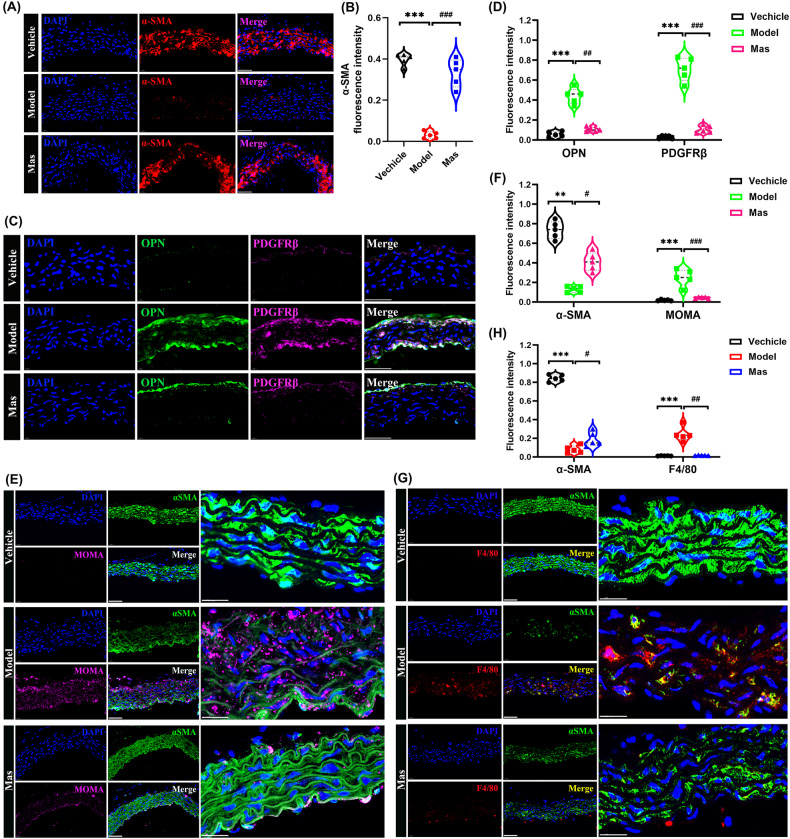
VSMCs dedifferentiated to macrophage-like switching phenotype. **A** Representative immunofluorescence images for detecting α-SMA (red), DAPI (blue) (*n* = 5 per group). Scale bar: 50 μm. **B** α-SMA fluorescence intensity. **C** Representative immunofluorescence images for double-labeling of OPN (green) and PDGFRβ (magenta) of aorta, DAPI (blue) (*n* = 5 per group). Scale bar: 50 μm. **D** OPN and PDGFRβ fluorescence intensity. **E** Representative immunofluorescence images for double-labeling of α-SMA (green) and MOMA (magenta) expressions of aorta, DAPI (blue) (*n* = 5 per group). Scale bar: 50 μm (low magnification) and 20 μm (high magnification). **F** α-SMA and MOMA fluorescence intensity. **G** Representative immunofluorescence images for double-labeling of αSMA (green) and F4/80 (red) expressions of aorta, DAPI (blue) (*n* = 5 per group). Scale bar: 50 μm (low magnification) and 20 μm (high magnification). **H** α-SMA and F4/80 fluorescence intensity. One-way ANOVA was followed by *post hoc* Tukey’s test. All results are presented as mean ± SEM. **P < 0.01,***P < *0.001 vs* vehicle group, ^#^*P* < *0.05*, ^#*#*^*P* < *0.01*, ^#*##*^*P* < *0.001, vs* model group.

As a kind of differentiated mature cells in human body, VSMCs can be expressed by a series of specific marker proteins, such as smooth muscle alpha-actin (α-SMA), which can be applied to distinguish cell identification and phenotypic status. Synthetic phenotype vascular smooth muscle cells (VSMCs) are mainly located in mid-embryonic or pathological blood vessels and express some markers of osteogenic differentiation, as osteopontin (OPN). The expression of the VSMCs differentiation markers (α-SMA) were significantly decreased (Fig. 2A, B), and the increased expression of VSMC dedifferentiation markers (OPN) co-expressed with PDGFRβ, as shown in Fig. 2C, D. In response to various pathological factors, differentiated VSMCs can give rise to SMC-derived fibrous cap cells, macrophage-like cells, mesenchymal stem cell like-cell, and osteochondrogenic cells [33]. As recent studies suggest that VSMCs dedifferentiate to macrophage-like cells and express macrophage foam cell markers, such as F4/80, MOMA-2. We further examined whether CAWS-induced macrophage-like dedifferentiated VSMCs. The colocalization of α-SMA and MOMA (Fig. 2E, F) or F4/80 (Fig. 2G, H) was further verified by double immunofluorescence analysis in CAWS-induced KD mice.

### Inducement of endothelial injury and VEGF levels increase in artery of KD murine model

**Fig. 3:**
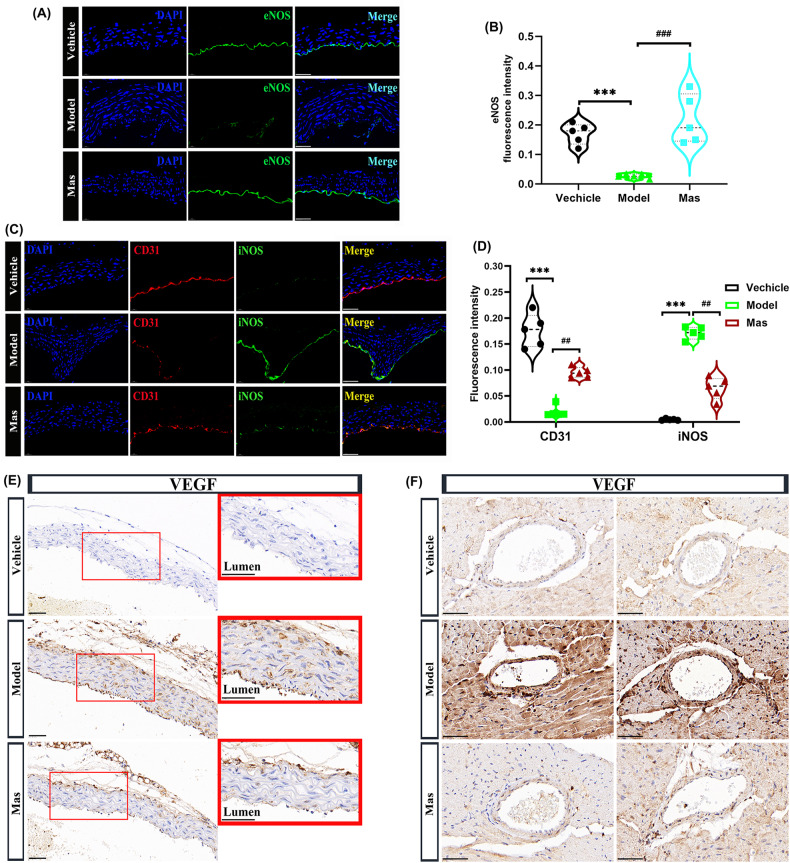
Inducement of arterial endothelial injury and VEGF levels increase in artery of KD murine model. **A** Representative immunofluorescence images for eNOS (green) expressions of aortic endothelium, DAPI (blue) (*n* = 5 per group). Scale bar: 50 μm. **B** eNOS fluorescence intensity. **C** Representative immunofluorescence images for double-labeling of iNOS (green) and CD31 (red) expressions of aorta, DAPI (blue) (*n* = 5 per group). Scale bar: 50 μm. **D** CD31 and iNOS fluorescence intensity. **E** Representative immumohistochemical images for VEGF expression in aorta (*n* = 4 per group). Enlarged images of area of interesting (AOI) were indicated with red block diagrams. Scale bar: 50 μm. **F** Representative immumohistochemical images for VEGF expression in coronary bifurcation arteritis (*n* = 4 per group). Scale bar: 50 μm. One-way ANOVA was followed by *post hoc* Tukey’s test. All results are presented as mean ± SEM.***P < *0.001 vs* vehicle group, ^#*#*^*P* < *0.01*, ^#*##*^*P* < *0.001 vs* model group.

Nitric oxide (NO)-driven modifications regulate a wide range of cellular processes and have been highlighted as an epigenetic event that protects proteins from proteolytic degradation. The production of NO by the family of NO synthase (NOS) isozymes, mediates many homeostatic and pathophysiologic responses [34]. The induction processes that stimulate expression of the inducible NOS isozyme (iNOS) have also been shown to downregulate the expression of the eNOS isozyme. As a major mediator of inflammation, iNOS plays an important role in cardiovascular pathophysiology through its elevated activity and inducement of excess NO production. The robust induction of iNOS within the aorta of KD mice full stands for the inflammatory injury. We examined endothelial function after the onset of KD, the dysfunction represented by the decrement of eNOS (Fig. 3A, B) and CD31 in KD model group, furthermore, the increasement of iNOS at the same time (Fig. 3C, D).

Among the pathologic findings of KD vasculitis, the increased vascular permeability plays a vital role, which leads to vascular leakiness, perivascular edema, decreased plasma proteins, and inflammatory cell infiltration of endothelium [14]. During the development of vascular leakage, VEC hyperpermeability induced by vasoactive factors has been shown the close association [35]. VEGF is the most well-described pathological hyperpermeability one, which induces the synthesis of interstitial collagenase, metalloproteinase, and plasminogen activators and the development of fenestration in the endothelium of small venules and capillaries. VEGF serum/plasma levels in KD patients increase in the acute or subacute stages [36], which may contribute to the leakage of substances in plasma, such as albumin, and the subendothelium infiltration of blood cells [37]. VEGF is also a potent chemoattractant for monocytes and induces expression of adhesion molecules on the endothelial cells. VEGF also enhances proliferation and migration of endothelial cells in collaboration with NO and may contribute to later vascular remodeling after the acute phase of KD [38, 39]. In this study, VEGF levels in aorta (Fig. 3E) and coronary artery (Fig. 3F) were examined. In comparison to vehicle mice, CAWS-induced mice presented remarkably elevated VEGF levels. Mas administration significantly reduced the VEGF expression in CAWS-triggered murine models of KD.

### Mas inhibits VECs migration through RhoA/ROCK signaling pathway in VECs

**Fig. 4:**
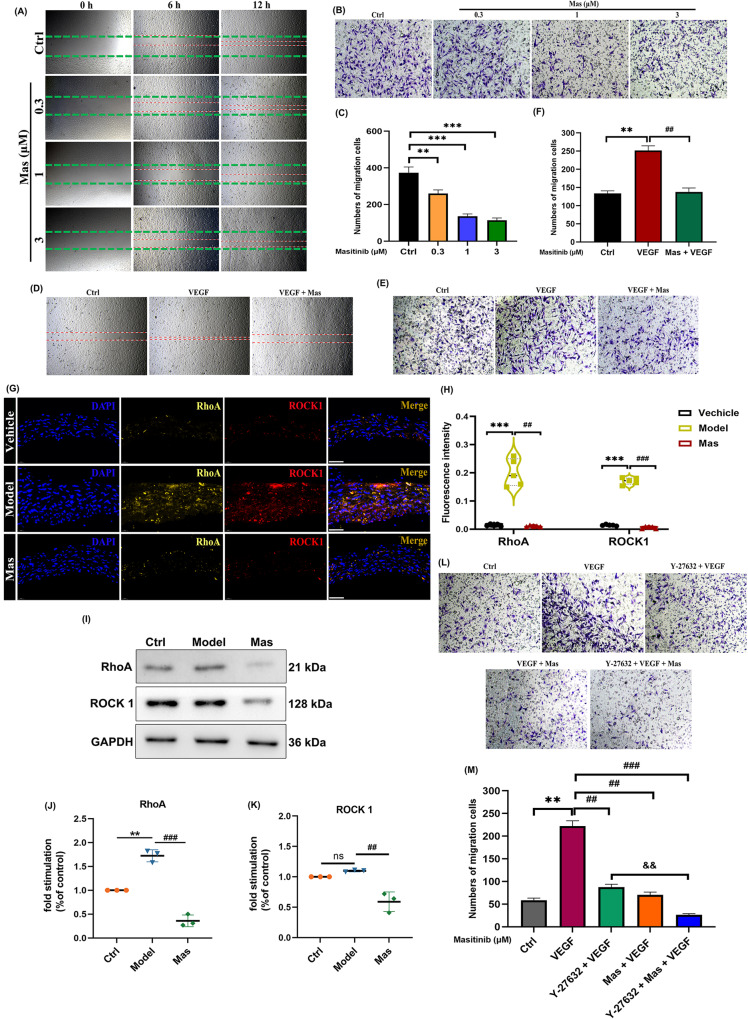
Mas inhibits VECs migration through RhoA/ROCK signaling pathway in VECs. **A** Representative images of wound healing assays. HUVECs had been exposed to different concentrations of Mas (0.3, 1, 3 μM) for 12 h. **B** Representative images of migrated HCAECs that had been exposed to different concentrations of Mas (0.3, 1, 3 μM). The migration capacity of HCAECs was determined using a Transwell culture system. **C** The quantification of migrated cells was counted from 5 different views. **D** Representative images of wound healing assays. HUVECs had been exposed to VEGF (1 nM), Mas (1 μM) + VEGF (1 nM) for 12 h. **E** Representative images of migrated HCAECs that had been exposed to VEGF (1 nM), Mas (1 μM) + VEGF (1 nM) for 12 h.The migration capacity of HCAECs was determined using a Transwell culture system. **F** The quantification of migrated cells was counted from 5 different views. Cells were observed at 40×magnification. **G** Representative immunofluorescence images for double-labeling of RhoA (yellow) and ROCK1 (red) expressions of aorta, DAPI (blue) (n = 5 per group). Scale bar: 50 μm (low magnification) and 20 μm (high magnification). **H** RhoA and ROCK1 fluorescence intensity. **I**–**K** Representative western blot analysis to determine the proteins expression of RhoA and ROCK1 in HUVEC cells. **L** In the absence or presence of 10 μM of ROCK inhibitor Y-27632, the migration capacity of HCAECs was determined using the Transwell culture system. **M** The quantification of migrated cells was counted from 5 different views. Cells were observed at 40×magnification. One-way ANOVA was followed by *post hoc* Tukey’s test. All results are presented as mean ± SEM. **P < 0.01,***P < *0.001 vs* ctrl group, ^#*#*^*P* < *0.01*, ^#*##*^*P* < *0.001 vs* VEGF group, ^&&^*P* < *0.01 vs* VEGF + Y-27632 group.

Microvasculature, as an ubiquitous organ system, is known to play a major role in the pathogenesis of cardiovascular injury. VECs that form the inner lining of microvessels, are critical targets for stimuli or agents and are responsible of stressor-induced acute vascular dysfunctions. Migration and proliferation of endothelial cells are involved in re-endothelialization and angiogenesis, two important cardiovascular processes. VEGF promotes VECs survival, proliferation, permeability, and taxis toward the VEGF source, essential steps in the production and maintenance of blood vessels. Considering the high levels of VEGF in arterial endothelial cells, the migration of VECs was next examined. We applied both HUVEC and HCAEC cells to evaluate the effect of Mas on cell migration. As shown in Fig. 4A–C, treatment with different concentrations of Mas inhibited the migration of VECs, while 1 μM of Mas exhibited the best inhibitory effect. Furthermore, the migration of VECs induced by VEGF also been inhibited by Mas (Fig. 4D–F).

RhoA is a member of the Ras superfamily of small GTP binding proteins that regulate formation of actin stress fibers and focal adhesions. Rho-Kinases (including ROCK1 and ROCK2) are the downstream effect factors of RhoA. RhoA/ROCK signaling plays important regulatory roles in many intracellular processes including cell proliferation, differentiation, and migration. In this study, we examined the activation of this pathway. The colocalization of high levels of RhoA and ROCK in aorta of CAWS-triggered KD murine models demonstrated the activation of this pathway. Mas administration significantly reduced this inappropriate activation (Fig. 4G, H). Besides, we used HUVEC cells to evaluate the effect of Mas on RhoA/ROCK1 pathway. Mas inhibited the expression of RhoA and ROCK1 (Fig. 4I–K). ROCK inhibitor Y-27632 was applied to prevent this pathway activation. The migration of VECs induced by VEGF could also be inhibited by Y-27632, and the inhibition was more significant when Y-27632 and Mas were used in combination (Fig. 4L, M).

### DRP-1-dependent mitochondrial dysfunction caused by dysregulated fission in the artery of KD murine model

**Fig. 5:**
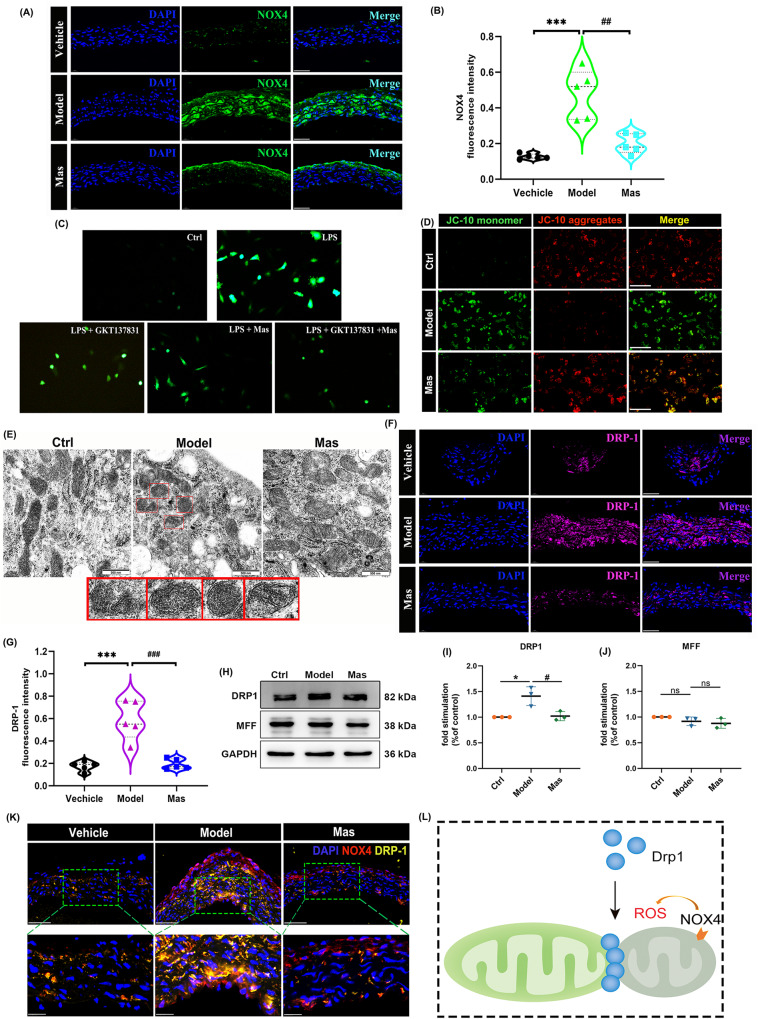
DRP-1-dependent mitochondrial dysfunction caused by dysregulated fission in the artery of KD murine model. **A** Representative immunofluorescence images for detecting NOX4 (green) expressions within aorta, DAPI (blue) (*n* = 5 per group). Scale bar: 50 μm. **B** NOX4 fluorescence intensity. **C** HUVECs were pre-treated with 10 μM of NOX4 inhibitor GKT137831 or Mas for 2 h, then LPS for 24 h. Dihydroethidium (DHE; 10 mM) was added to cells for another 30 min. Pictures were collected. Cells were observed at 100 × magnification. **D** Mitochondrial membrane potential was measured by using JC-10 dye. Graphical representation of the ratio of JC-1 aggregates to JC-10 monomers. Scale bars, 20 μm. **E** The appearance of mitochondria was observed by TEM. Mitochondrial fragmentation was shown in enlarge image in red frame. **F** Representative immunofluorescence images for DRP-1 (magenta) expressions of aortic endothelium, DAPI (blue) (*n* = 5 per group). Scale bar: 50 μm. **G** DRP-1 fluorescence intensity. **H**–**J** HUVECs were pre-treated with Mas (3 μM) for 2 h and then, LPS was applied to induce inflammatory injury together with Mas for another 48 h. Model group was treated with LPS only for 48 h. Representative western blot analysis to determine the proteins expression of DRP-1 and MFF in HUVEC cells. **K** Representative immunofluorescence images for double-labeling of NOX4 (red) and DRP-1 (yellow) expressions of aorta, DAPI (blue) (*n* = 3 per group). Scale bar: 50 μm. **L** DRP-1-dependent mitochondrial dysfunction was described in a graphical representation. One-way ANOVA was followed by *post hoc* Tukey’s test. *P < *0.05, vs* vehicle group. ***P < *0.001 vs* vehicle or Ctrl group, ^*#*^*P* < *0.05*, ^*##*^*P* < *0.01*, ^#*##*^*P* < *0.001 vs* model group.

Reactive oxygen species (ROS) are produced in all cell types of the vasculature, including endothelial cells, smooth muscle cells, and so on. ROS, including the superoxide anion, the hydroxyl radical, and hydrogen peroxide, are critical signaling molecules with important roles in both cardiac physiology and disease. From the source, cytosolic part, including NADPH oxidases (NOX), xanthine oxidase, cyclooxygenases, and cytochrome P450 enzymes, and mitochondrial part, including the respiratory chain, monoamine oxidases (MAOs), p66shc, and NOX4, contribute to the intracellular ROS pool [40]. In stressed or pathological conditions, the local ROS concentration results from the interplay of production by ROS-generating enzymes, such as NOX, leading to increased bioavailability of ROS (termed oxidative stress) [41]. NOX are a key source of oxidative stress in human arteries, the NOX4-dependent effects that induced vascular dysfunction may mirror the role of this homolog in heart failure [42]. The elevated ROS levels can result in activation of the mitochondrial permeability transition pore (MPTP), mitochondrial dysfunction, and cell death. Here, we explored that NOX4 were upregulated in CAWS-triggered KD murine models and Mas reversed this increase (Fig. 5A, B). Microscopic analysis demonstrated a significant increase of ROS by LPS. Mas or NOX4 inhibitor GKT137831 can inhibit this production of ROS (Fig. 5C). JC-10 dye concentrates in mitochondrial matrix and forms red fluorescent aggregates in normal cells as a result of the existence of the electrochemical potential shift from red (JC-10 aggregates) to green fluorescence (JC-10 monomers). Hypertrophied cells exhibited depolarized ΔΨm, which was evident from the significantly higher number of JC-10 monomers (green fluorescence). The increased ratio of green staining after LPS treatment indicated a lower mitochondrial membrane potential in VECs. The ratio of JC-1 aggregates to JC-1 monomers was recorded (Fig. 5D). Next, the LPS-induced endothelial injury model was applied to observed the mitochondria appearance by transmission electron microscopy (TEM). Ruptured mitochondria were detected in the model group, as shown in enlarge image in red frame, suggesting VECs injury. Mas treated could relieve the mitochondria dysfunction to lessen the VECs damage (Fig. 5E).

Mitochondrial quality control mechanisms include mitochondrial biogenesis, mitochondrial dynamics, and mitophagy. As the highly dynamic organelles, mitochondria undergo fusion and fission to regulate their morphology and control their number and size, this process called “mitochondrial dynamics” [43]. Emerging evidence suggests that disrupted mitochondrial dynamics play vital roles in the pathogenesis of many cardiovascular diseases (CVDs), mainly by influencing cellular energy, ROS generation, intracellular calcium levels, apoptogenic protein production, and some other mechanisms in a cell- or tissue-specific manner [44]. Mitochondrial dynamics processes are regulated by specific proteins, known as mitochondria-shaping proteins [45]. The cytosolic GTPase dynamin-related protein 1 (DRP-1) is the main pro-fission protein with activity that is tightly controlled to ensure balanced mitochondrial dynamics according to cellular needs [45]. All these findings indicate a close relationship between mitochondrial dynamics in CVDs and DRP-1-induced mitochondrial fragmentation. Since mitochondrial fragmentation is often observed when cells are under stress or when they are dying, DRP-1 has been implicated in the pathogenesis of cell death [46]. In this study, we demonstrated the overexpression of DRP-1 in aorta of KD murine model (Fig. 5F, G). When the DRP-1 was hyperexpression in the LPS-induced endothelial injury model, Mas administration could restrain DRP-1 content (Fig. 5H–J). NOX4-related DRP-1 hyperexpression was demonstrated by the colocalization of NOX4 and DRP-1 in aorta (Fig. 5K). This DRP-1-dependent mitochondrial dysfunction caused by NOX4-mediated mitochondria dysregulated fission was described in a graphical representation (Fig. 5L).

### Activation of HIF-1α/BNIP3 signaling pathway in artery of KD murine model

**Fig. 6:**
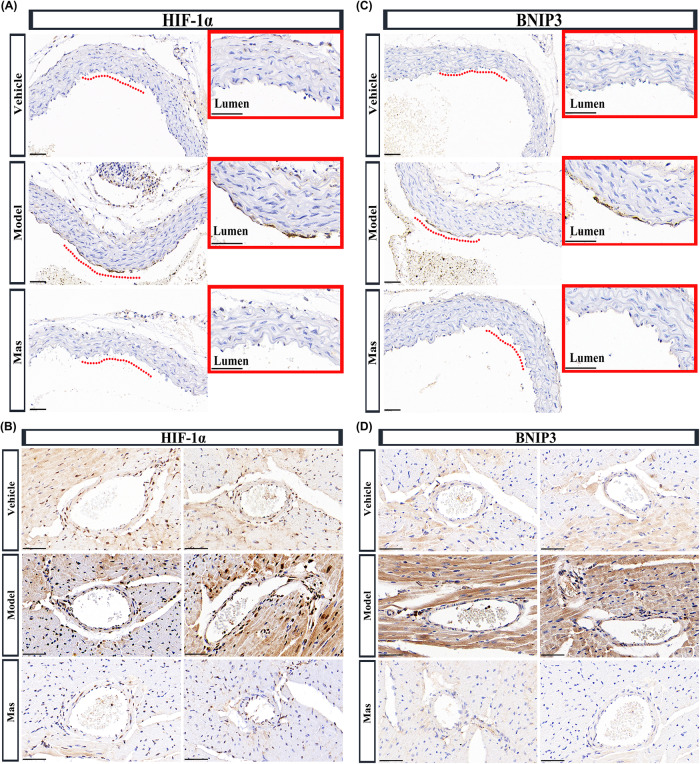
Activation of HIF-1α/BNIP3 signaling pathway in artery of KD murine model. **A** Representative immumohistochemical images for HIF-1α expression in aorta (*n* = 4 per group). Enlarged images of area of interesting (AOI) were indicated with red block diagrams. Scale bar: 50 μm. **B** Representative immumohistochemical images for HIF-1α expression in coronary bifurcation arteritis (*n* = 4 per group). Scale bar: 50 μm. **C** Representative immumohistochemical images for BNIP3 expression in aorta (*n* = 4 per group). Enlarged images of area of interesting (AOI) were indicated with red block diagrams. Scale bar: 50 μm. **D** Representative immumohistochemical images for BNIP3 expression in coronary bifurcation arteritis (*n* = 4 per group). Scale bar: 50 μm.

Mitochondria are maternally inherited multifunctional organelles that can rapidly adjust to meet the metabolic needs of the cell. They play a vital role in bioenergetic and biosynthetic pathways by an intricate balance between fission and fusion, mitochondrial biogenesis, and mitophagy. Mitochondrial damage can induce ROS production, and ROS oxidative stress can regulate mitochondrial autophagy by regulating hypoxia inducible factor 1α (HIF-1α). Bcl-2 19-kDa interacting protein 3 (BNIP3) localizes to mitochondria when overexpressed, thus, BNIP3 is a proapoptotic protein that may function through a mitochondrial pathway. It has been confirmed that BNIP3 is the target molecule of HIF-1α induced by hypoxia or ischemia, and subsequently induces mitochondrial autophagy. In this study, we demonstrated the high expression of HIF-1α in aorta (Fig. 6A) and coronary artery (Fig. 6B) of KD model mice. At the same time, the BNIP3 up-regulation was demonstrated in aorta (Fig. 6C) and coronary artery (Fig. 6D).

### DRP-1-dependent segregation of damaged mitochondria promotes mitophagy for degradation

**Fig. 7:**
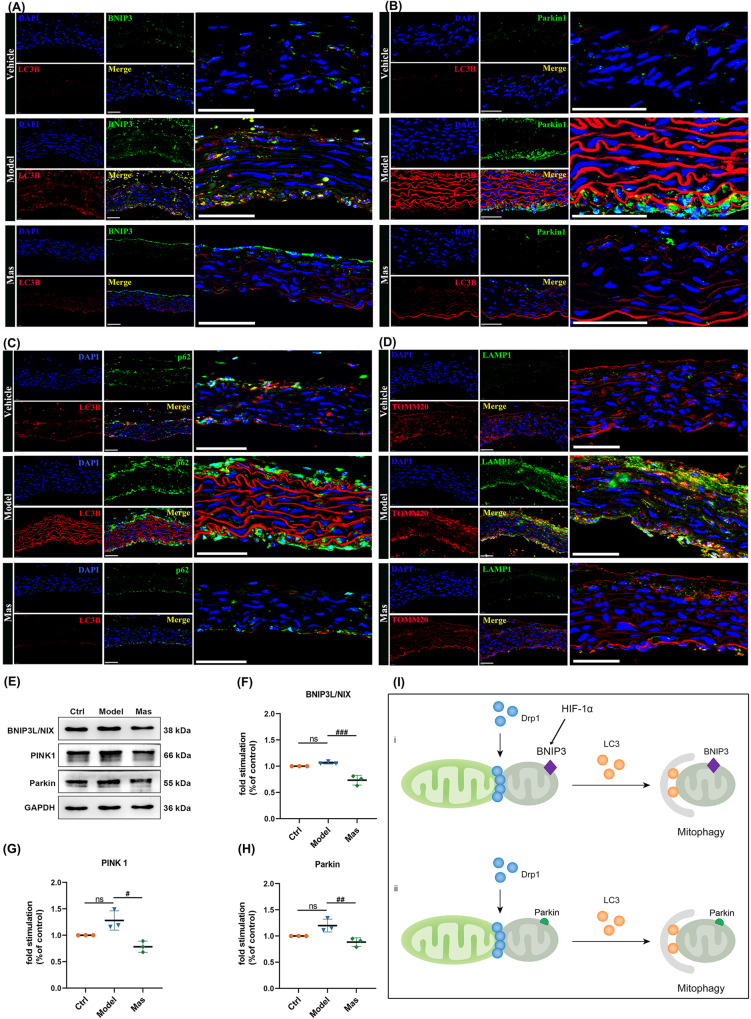
DRP-1-dependent segregation of damaged mitochondria promotes mitophagy for degradation. **A** Representative immunofluorescence images for colocalization of BNIP3 (green) and LC3B (red) expressions of aorta, DAPI (blue) (*n* = 4 per group). Scale bar: 50 μm. **B** Representative immunofluorescence images for colocalization of Parkin (green) and LC3B (red) expressions of aorta, DAPI (blue) (*n* = 4 per group). Scale bar: 50 μm. **C** Representative immunofluorescence images for double-labeling of p62 (green) and LC3B (red) expressions of aorta, DAPI (blue) (*n* = 4 per group). Scale bar: 50 μm. **D** Representative immunofluorescence images for double-labeling of LAMP1 (green) and TOMM20 (red) expressions of aorta, DAPI (blue) (*n* = 4 per group). Scale bar: 50 μm. **E**–**H** HUVECs were pre-treated with Mas (3 μM) for 2 h and then, LPS was applied to induce inflammatory injury together with Mas for another 48 h. Model group was treated with LPS only for 48 h. Representative western blot analysis to determine the proteins expression of BNIP3L/Nix, PINK1, and Parkin in HUVEC cells. One-way ANOVA was followed by *post hoc* Tukey’s test. ^#^*P* < *0.05*, ^#*#*^*P* < *0.01*, ^#*##*^*P* < *0.001, vs* model group. **I** DRP-1-dependent segregation of damaged mitochondria promoting mitophagy for degradation was described in a graphical representation.

It is generally believed that healthy mitochondria can restore mitochondrial membrane potential levels and participate in the fusion-fission cycle, whereas mitochondria that fail to do so are marked and eliminated by mitophagy. Elimination of damaged mitochondria by mitochondria-targeted selective autophagy, “mitophagy”. If damage accumulates in mitochondria, the mitochondria are aggregated and segregated by fission, followed by elimination of the damaged mitochondria via mitophagy. Considering the essential role of DRP-1 in mediating mitochondrial fission and the overexpression in the aorta of our KD murine model, we next examined the mitophagy level of artery. We confirmed the high expression of BNIP3 and colocalization with LC3B (Fig. 7A). DRP-1 promotes mitophagy in many cell types in response to different types of stress. It has been reported that inhibition of DRP-1-mediated mitochondrial fission prevents Parkin-induced mitophagy in HeLa cells [47]. The BNIP3 induces mitochondrial translocation of DRP-1, which in turn promotes translocation of Parkin to mitochondria in a DRP-1-dependent manner in adult cardiomyocytes [48]. It follows then that DRP-1 is required for BNIP3-induced mitophagy by inducing mitochondrial fission and recruitment of Parkin in a coordinated manner [49]. Double immunofluorescent staining revealed colocalization of LC3B and Parkin proteins in the aorta of KD model murine (Fig. 7B). The level of mitophagy was examined by double-labeled with LC3B and p62 (Fig. 7C), meanwhile LAMP1 and TOMM20 (Fig. 7D). The excessive mitophagy within the aorta of KD mice without suspense. The LPS-induced endothelial injury model was also applied to observed the mitophagy. In model group, the BNIP3L/NIX, PINK1 and Parkin were increased in varying degrees, and Mas could inhibit these upregulations remarkably (Fig. 7E–H). DRP-1-dependent segregation of damaged mitochondria promotes mitophagy for degradation was described in a graphical representation (Fig. 7I).

### DRP-1 associates with Bak at mitochondrial fission sites to induce cytochrome c release

**Fig. 8:**
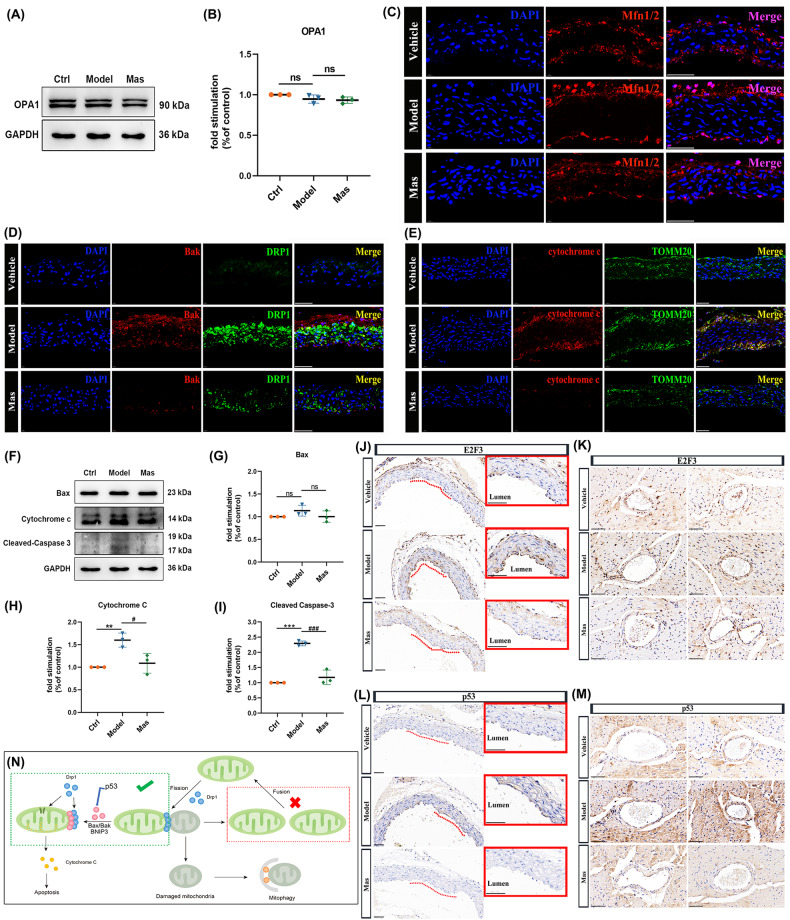
DRP-1 associates with Bak at mitochondrial fission sites to induce cytochrome c release. **A**, **B** HUVECs were pre-treated with Mas (3 μM) for 2 h and then, LPS was applied to induce inflammatory injury together with Mas for another 48 h. Model group was treated with LPS only for 48 h. Representative western blot analysis to determine the proteins expression of OPA1 in HUVEC cells. **C** Representative immunofluorescence images for detecting Mfn1/2 (red), DAPI (blue) (*n* = 4 per group). Scale bar: 50 μm. **D** Representative immunofluorescence images for colocalization of DRP-1 (green) and Bak (red) expressions of aorta, DAPI (blue) (*n* = 4 per group). Scale bar: 50 μm. **E** Representative immunofluorescence images for colocalization of TOMM20 (green) and cytochrome c (red) expressions of aorta, DAPI (blue) (*n* = 4 per group). Scale bar: 50 μm. **F**–**I** HUVECs were pre-treated with Mas (3 μM) for 2 h and then, LPS was applied to induce inflammatory injury together with Mas for another 48 h. Model group was treated with LPS only for 48 h. Representative western blot analysis to determine the proteins expression of Bax, cytochrome c and cleaved-caspase 3 in HUVEC cells. **J** Representative immumohistochemical images for E2F3 expression in aorta (n = 4 per group). Enlarged images of area of interesting (AOI) were indicated with red block diagrams. Scale bar: 50 μm. **K** Representative immumohistochemical images for E2F3 expression in coronary bifurcation arteritis (*n* = 4 per group). Scale bar: 50 μm. **L** Representative immumohistochemical images for p53 expression in aorta (*n* = 4 per group). Enlarged images of area of interesting (AOI) were indicated with red block diagrams. Scale bar: 50 μm. **M** Representative immumohistochemical images for p53 expression in coronary bifurcation arteritis (*n* = 4 per group). Scale bar: 50 μm. **N** The schematic diagram describes the mitophagy which seems to work as the driving force of DRP-1/Bak/BNIP3-dependent endothelial cells apoptosis. One-way ANOVA was followed by *post hoc* Tukey’s test. ^**^P < *0.01*, ^***^P < *0.001*, *vs* ctrl group, ^#^*P* < *0.05*, ^###^*P* < *0.001*, *vs* model group.

Mitochondrial division creates small mitochondria, and mitochondrial fusion produces large mitochondria. Under pathological conditions, significantly larger or smaller mitochondria have been observed. It has been reported that mitophagy involves DRP-1, which generates small mitochondria and allows autophagosomes to efficiently engulf the organelle. Too much mitochondrial fission negatively affects mitochondrial function, stimulating mitochondrial biogenesis could compensate for the deleterious effects [50]. Comprehensive consideration of literature reports of the protective role of mitophagy, we presumed that the healthy mitochondrial fraction after mitochondrial fission then fused each other, allowing mitochondria to compensate for one another’s defects, maintain the membrane potential, complement protein components, and complete mtDNA repair. However, OPA1 (Fig. 8A, B) and mitochondrial fusion protein (Mfn1/2) expression (Fig. 8C), in LPS-induced endothelial injury model or aorta of KD murine model were free from influence, under Mas treatment or not. It has been reported that DRP-1 associated with Bax/Bak at mitochondrial fission sites during apoptosis in HeLa cells [51] and controlled cell death by acting downstream of Bax/Bak translocation but upstream of cytochrome c release [52]. The combination of Bak with DRP-1 was examined by double-labeling immunofluorescence (Fig. 8D), and downstream cytochrome c release (Fig. 8E). In LPS-induced endothelial injury model, the cytochrome c and cleaved caspase 3 were upregulated obviously and Mas could reverse this increasement (Fig. 8F–I).

Many studies have demonstrated that E2F family transcription factors play important regulatory roles in the cardiovascular system. It is closely related to the function of vascular endothelial cells and cardiomyocytes, whose dysfunction may be associated with the pathogenesis of various cardiovascular diseases. Among this factors, E2F3 knockout mice have dysplasia or congestive heart failure, indicating that E2F3 plays an important role in the development of the cardiovascular system or maintenance of its normal functions. In addition to inducing cellular proliferation, the overexpression of E2F can also trigger cells to undergo apoptosis. There is considerable evidence to suggest that this can occur through both p53-dependent and p53-independent mechanisms [53, 54]. E2F3 is a mediator of DNA damage-induced apoptosis [55]. In this study, we examined the E2F3 expression in aorta and small coronary artery. In comparison to vehicle mice, CAWS-induced mice presented elevated E2F3 levels in VECs. Mas administration significantly reduced the E2F3 expression in CAWS-triggered murine models of KD (Fig. 8J, K). The high expression of E2F3 induced p53 activation of endothelial cells (Fig. 8L, M). Here, the mitophagy seems to work as the driving force of DRP-1/Bak/BNIP3-dependent endothelial cells apoptosis, shown in the schematic diagram (Fig. 8N).

## Discussion

Kawasaki disease (KD) is an acute inflammatory syndrome of unknown etiology that is now a common cause of acquired heart disease in children. Chronic inflammation (vasculitis) due to KD might cause vascular cellular senescence and vascular endothelial cell damage, and is a potential cause of myocardial ischemia in young adults in young adults [56]. Coronary artery involvement is considered a specific criterion supportive of the diagnosis of KD, particularly for those patients who do not meet the full clinical criteria for a diagnosis of complete KD. The coronary artery abnormalities associated with KD can be differentiated from lesser degrees of dilation that may be rarely present with other febrile illnesses. Furthermore, several non-coronary complications have also been identified in this condition [7]. Pathological outcomes of coronary artery damage depend on the severity of the lesions. During KD coronary vasculopathy, the microenvironment of the damaged area is changed and inflammatory response is activated. Compared with the vehicle group, the spleen of our CAWS-induced KD model mice was enlarged and black in color (Fig. 1B). We examined the serum levels of pro-inflammatory cytokines, CAWS-induced mice presented remarkably elevated IL-1β, IL-6, IL-18 and TNF-α levels (Fig. 1C–F). In our CAWS-induced KD mice model, the typical pathological changes of KD vasculitis and cardiac injury were successfully induced (Fig. 1I–M). Considering the vital role of PDGF in pathogenesis of vascular damage in KD, in this experiment, the expression of its receptor, PDGFRβ was examined. Hyperexpression of PDGFRβ was induced in the thickened medial layer of CAWS-induced KD mice (Fig. 1G, H). Furthermore, VSMCs dedifferentiated to macrophage-like cells, with the reduced expression of the VSMCs differentiation markers (α-SMA) (Fig. 2A, B), and the increased expression of VSMC dedifferentiation markers (OPN) co-expressed with PDGFRβ (Fig. 2C, D), and expressed macrophage foam cell markers, such as MOMA-2 (Fig. 2E, F), F4/80 (Fig. 2G, H). Mas, the inhibitor of PDGFRβ, could ameliorate these cardiac and vascular inflammation.

When the vascular structure is damaged, the inner cortex is destroyed firstly. KD-like vasculitis impaired vascular endothelial cells that produce eNOS, which maintains vascular homeostasis, and promoted macrophage infiltration into the tissue [57]. eNOS, which is expressed specifically in vascular endothelial cells, is primarily associated with the production of the vasoactive substance NO, and is an important factor when assessing endothelial cell function. CAWS vasculitis has also been speculated to induce cellular senescence of vascular endothelial cells, leading to suppression of eNOS. Previous studies have shown that eNOS is activated via the Rho/ROCK pathway. Inhibition of Rho/ROCK pathway can restrain eNOS expression. The induction processes that stimulate expression of the inducible NOS (iNOS) have also been shown to downregulate the expression of the eNOS [34]. As a major mediator of inflammation, iNOS plays an important role in cardiovascular pathophysiology through its elevated activity and inducement of excess NO production. We examined endothelial function after the onset of KD, the dysfunction represented by the decrement of eNOS and CD31 in KD model group, furthermore, the increasement of iNOS at the same time (Fig. 3A–D). It has been reported that VEGF is an important factor in mediating the inflammation of KD. The literature regarded the relationship between VEGF and KD very exhaustively. KD patients with acute coronary artery lesions (CALs) had higher median VEGF levels than those without acute CALs from acute to convalescent phases. In the subacute phase, KD patients with acute CALs had significantly higher VEGF levels than those without acute CALs. VEGF did not decrease after IVIG treatment, and increased significantly after IVIG treatment in KD patients with acute CALs in acute phase. VEGF might be related to the complications of CALs in KD patients [13]. However, VEGF delivery alone may act as a negative regulator of vessel maturation [58], which is determined by pericyte recruitment mainly controlled by PDGF-BB [59]. Moreover, a recent clinical study shows a greater therapeutic efficacy by dual antagonism of PDGF and VEGF in treating age-related macular degeneration. PDGF in the tumor microenvironment activates PDGF receptor in ECs, which in turn induces NF-κB-dependent Snail expression, thereby inducing endothelial-mesenchymal transformation [60]. The roles of VEGF in acute KD may also involve promotion of vascular permeability and macrophage activation. VEGF also enhances proliferation and migration of endothelial cells in collaboration with NO and may contribute to later vascular remodeling after the acute phase of KD [14, 61]. In this study, VEGF levels in aorta and coronary artery were examined. In comparison to vehicle mice, CAWS-induced mice presented remarkably elevated VEGF levels. Mas administration significantly reduced the VEGF expression in CAWS-triggered murine models of KD (Fig. 3E, F). Furthermore, we demonstrated that treatment with different concentrations of Mas inhibited the migration of VECs (Fig. 4A–C), and VEGF-induced VECs migration (Fig. 4D–F) by inhibiting the activation of RhoA/ROCK1 pathway (Fig. 4G–M). The source of ROS in phagocytes is the NOX, an enzyme complex composed of membrane-bound and cytosolic subunits. NOX is a key source of oxidative stress in human arteries, for example, NOX1 is responsible for angiotensin II- and PDGF-stimulated ROS- production in rat aortic VSMCs [62]. The NOX4-dependent effects that induced vascular dysfunction may not mirror the role of this homolog in heart failure [42]. Here, we explored that NOX4 were upregulated in CAWS-triggered KD murine models and Mas reversed this increase (Fig. 5A, B). Microscopic analysis demonstrated a significant increase of ROS by LPS in HUVECs. Mas or NOX4 inhibitor GKT137831 can inhibit this production of ROS (Fig. 5C). JC-10 dye indicated a lower mitochondrial membrane potential by LPS (Fig. 5D) and mitochondrial morphological change examined by TEM (Fig. 5E).

Mitochondria exist as a dynamic coordinated network. In addition to ATP production, mitochondria regulate cell death and survival by integrating a range of cellular signals are also the primary source of ROS, which can trigger oxidative stress, thereby affecting cell survival and death. They maintain a healthy pool by constant fusion and fission [63], repair, sequestration or degradation via mitophagy or mitophagy-independent mechanisms [64], and biogenesis. DRP-1-mediated mitochondrial dynamics play crucial roles in mitochondrial quality control in the heart. It has been reported that the detrimental effects of excessive mitochondrial fission can be reversed by drugs targeting DRP-1 [43]. In this study, the DRP-1 was excessively expressed in the aorta of KD murine model and in the LPS-induced endothelial injury model. Mas administration could target DRP-1 to inhibit mitochondrial fragmentation (Fig. 5F–J). NOX4-related DRP-1 hyperexpression was demonstrated by the colocalization of NOX4 and DRP-1 in aorta (Fig. 5K). Mitochondrial damage can induce ROS production, and ROS oxidative stress can regulate mitochondrial autophagy by regulating HIF-1α. The activation of ROS-HIF-1α pathway promotes pulmonary artery smooth muscle cells proliferation, ultimately leads to pulmonary vascular remodeling [65]. It has been confirmed that BNIP3 is the target molecule of HIF-1α induced by hypoxia or ischemia, and subsequently induces mitochondrial autophagy [66]. Within the aorta and coronary artery of KD model mice exhibited upregulated levels of HIF-1α (Fig. 6A, B), downstream BNIP3 (Fig. 6C, D), and LC3B, as well as enhanced colocalization of LC3B with BNIP3 (Fig. 7A) and the activation of Parkin/LC3B pathway (Fig. 7B). The colocalization of LC3B with p62 (Fig. 7C), mitochondria with lysosomes (Fig. 7D), came down to hyper-mitophagy. Mitophagy, the selective engulfment of dysfunctional mitochondria by autophagasomes, is important for cellular homeostasis and can be induced by mitochondrial oxidative stress. As reported in the literature, DRP-1-dependent mitophagy can be triggered secondary to mitochondrial dysfunction to remove damaged mitochondria. The frequency of division and fusion is balanced to maintain mitochondrial size in healthy cells. Increased demand is met by mitochondrial biogenesis and fusion of individual mitochondria into dynamic networks, whereas a decrease in demand results in the removal of superfluous mitochondria through fission and mitophagy [67, 68]. However, mitochondrial fusion protein (Mfn1/2) and OPA1 expression, in aorta of KD murine model or LPS-induced endothelial injury model were free from influence, under Mas treatment or not (Fig. 8A–C). Since mitochondrial fragmentation is often observed when cells are under stress or when they are dying, DRP-1 has been implicated in the pathogenesis of cell death [46]. DRP-1 is involved in Bax/Bak- or BNIP3-dependent apoptosis by controlling outer mitochondrial membrane permeabilization [49]. Here, DRP-1 contacted with Bak in the fission site to induce cytochrome c release, then cell apoptosis (Fig. 8D–F). In addition, the high expression of E2F3 (Fig. 8J, K) induced p53 activation of endothelial cells (Fig. 8L, M). Surprisingly, the mitophagy seems to work as the driving force of DRP-1/Bak/cytochrome c-dependent endothelial cells apoptosis. DRP-1 overexpressing cell, split mitochondrial network by forced division.

Intact mitochondrial dynamics are critical for the maintenance of normal vascular function, and pharmacological modulation of mitochondrial dynamic proteins may emerge as novel therapeutic targets for a number of cardiovascular diseases resulting from excessive fission. However, targeting mitochondrial fission is a double-edged sword, since physiological mitochondrial fission under stress is necessary for the adaptation to increased energy demands, and repression of mitochondrial fission is not always beneficial. In this scenario, the process of mitochondrial fusion/fission emerges as an important regulator of mitochondrial signaling properties and of the broadly different intracellular effects of physiological and pathological stimuli. Mitochondrial homeostasis should be maintained when targeting mitochondrial dynamics under pathological conditions. Take a comprehensive view of the whole text, how mitophagy is regulated by DRP-1 under pathologic status is critical and complex, which may contribute to the development of specific therapeutic interventions in cardiovascular diseases patients, for example Masatinib, the inhibitor of PDGFRβ.

### Supplementary information

Original Western Blots

Fig.S1

Figure S1 legend

aj-checklist

## Data Availability

The data are available from the corresponding authors upon reasonable request.
