# Supplementary material for: Inhibition of PDGFRβ alleviates endothelial cell apoptotic injury caused by DRP-1 overexpression and mitochondria fusion failure after mitophagy
Source: Cell Death Dis. 2023 Nov 18;14(11):756. doi: 10.1038/s41419-023-06272-3 (PMC10657461; doi:10.1038/s41419-023-06272-3)

**Fig.4I**

**RhoA: Ctrl， LPS， MAS(3μM)+LPS, MAS(1μM)+LPS**

**Used:**


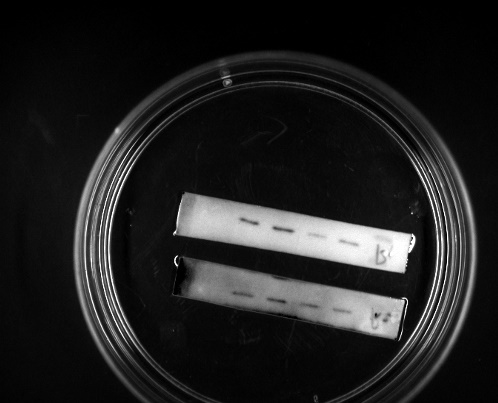




**Repeated:**


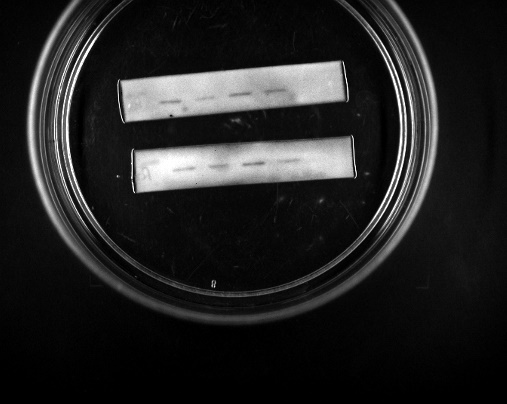




**ROCK1:**

**Used: MAS(1μM)+LPS, Ctrl， LPS， MAS(3μM)+LPS**


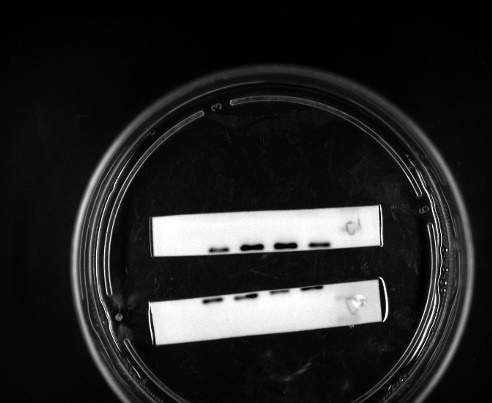




**Repeated: Ctrl， LPS， MAS(3μM)+LPS, MAS(1μM)+LPS**







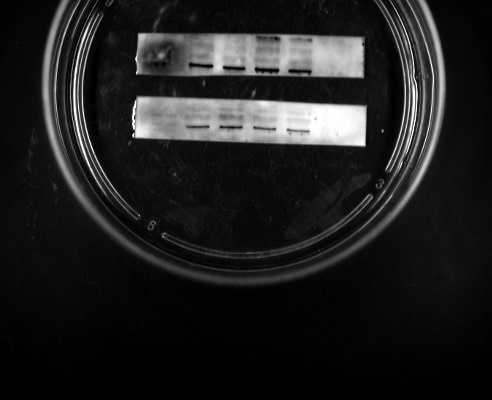






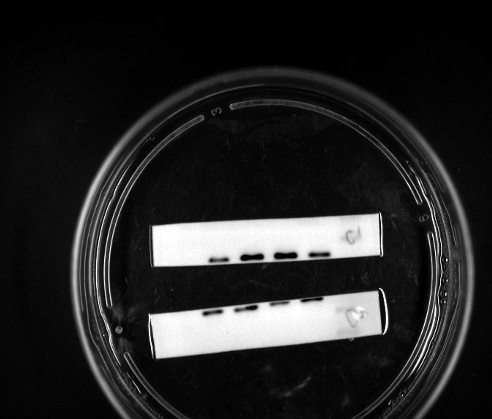


**Fig.5H**

**DRP1**

**（Used）：Ctrl LPS LPS+Mas-3μM**


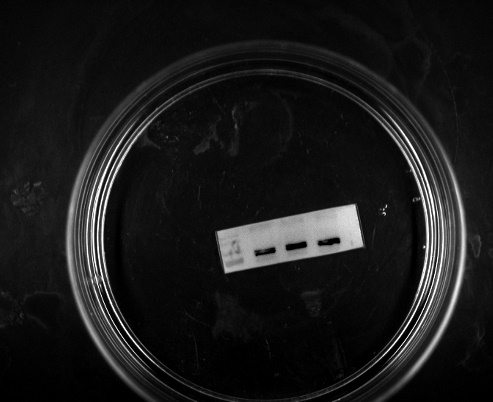




**（Repeated）：Ctrl LPS LPS+Mas-3μM**


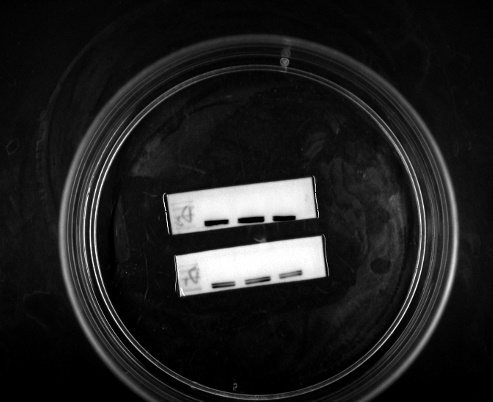




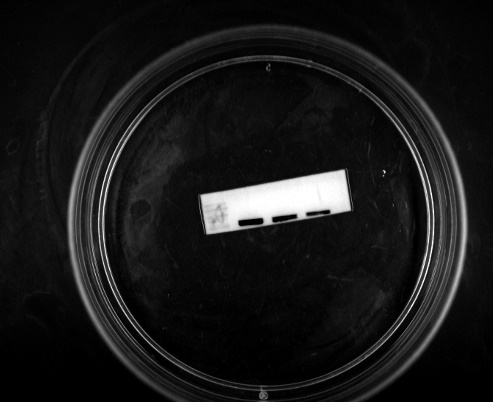




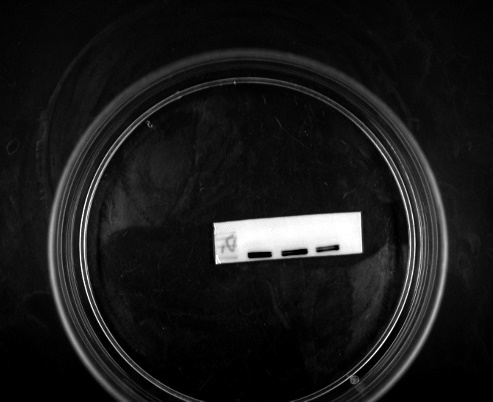




**MFF**

**（Used）：Ctrl LPS LPS+Mas-3μM**


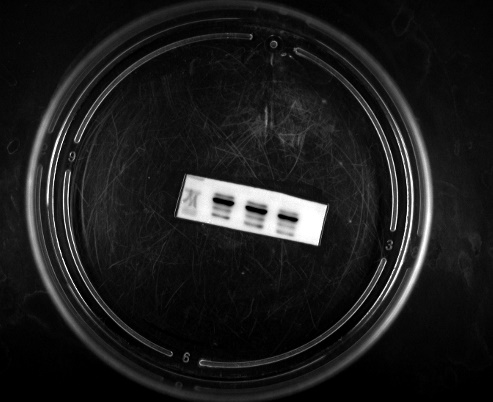




**（Repeated）：Ctrl LPS LPS+Mas-3μM**


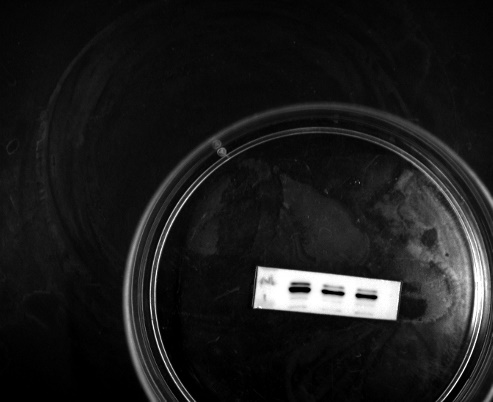




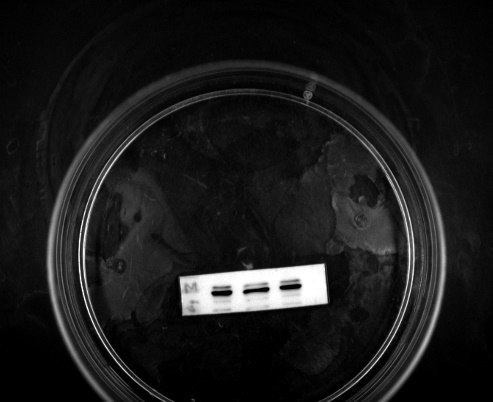




**Fig.7E**

**NIX**

**（Used）：Ctrl LPS LPS+Mas-3μM**


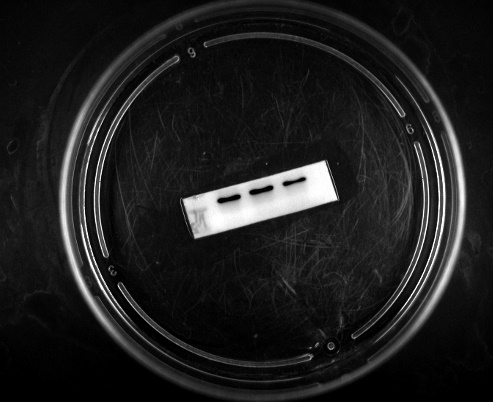




**（Repeated）：Ctrl LPS LPS+Mas-3μM**


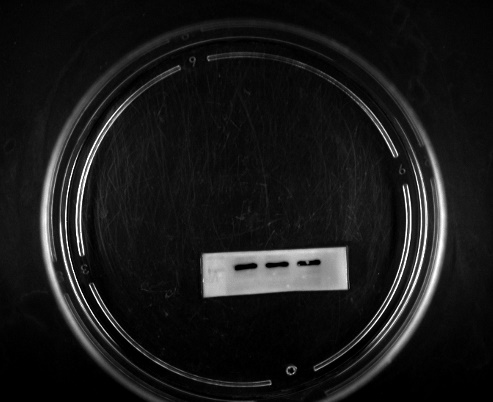




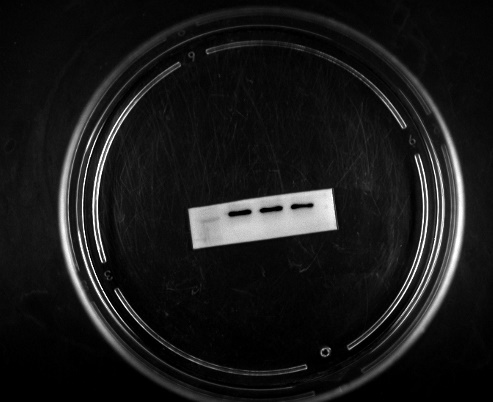




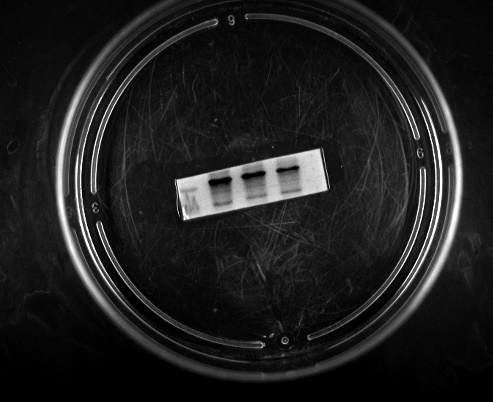




**PINK1**

**（Used）：Ctrl LPS LPS+Mas-3Μm**


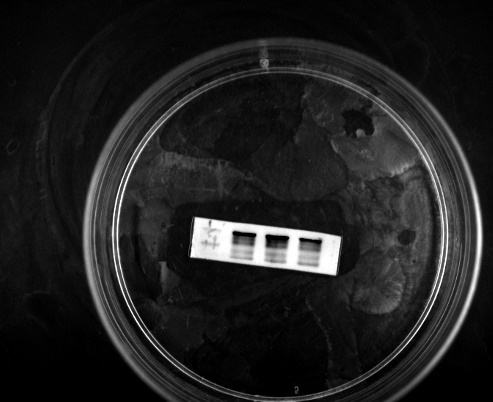




**（Repeated）：Ctrl LPS LPS+Mas-3μM**


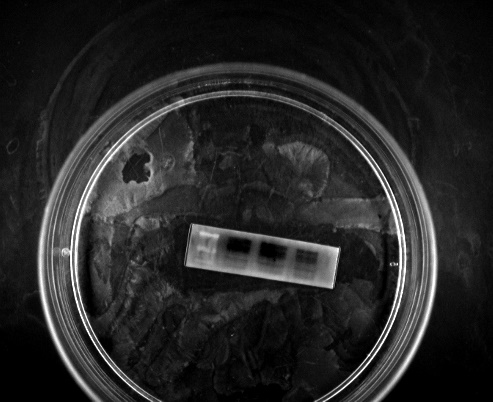




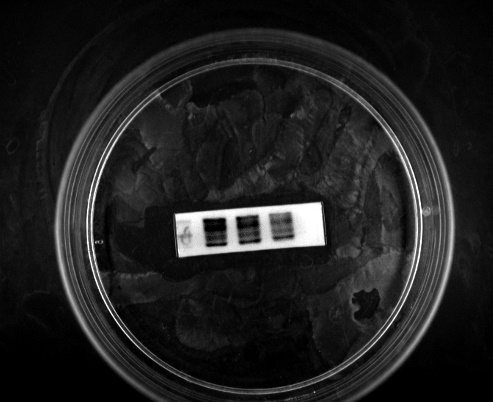




**parkin**

**（Used）：Ctrl LPS LPS+Mas-3μM**


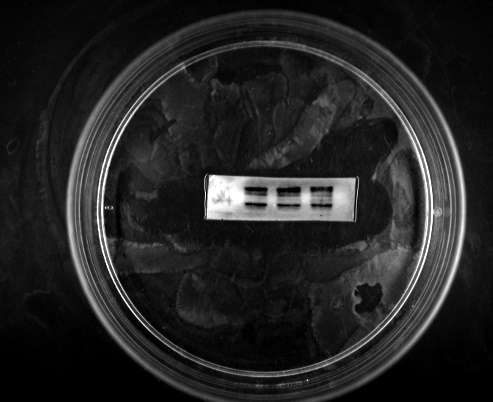




**（Repeated）：Ctrl LPS LPS+Mas-3μM**


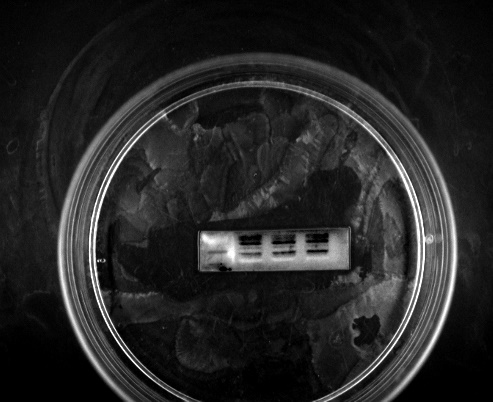




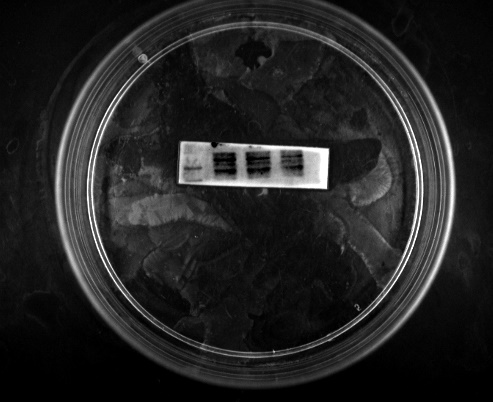




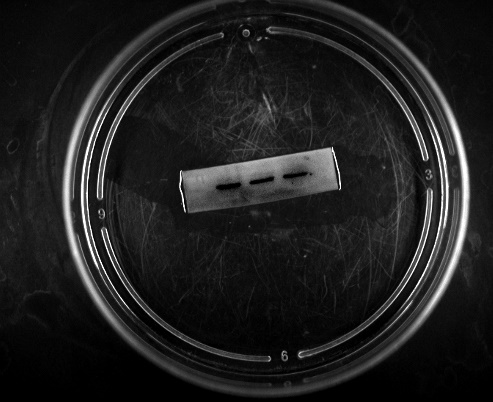




**Fig.8A**

**OPA1**

**（Used）：Ctrl LPS LPS+Mas-3μM**


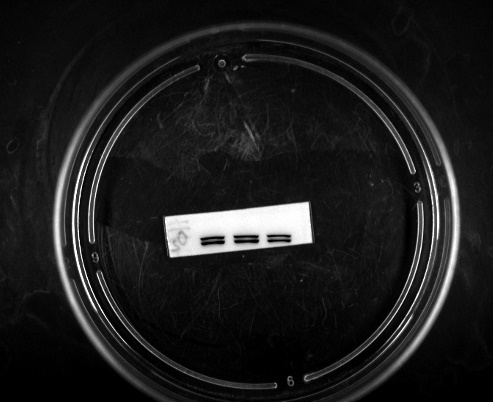




**（Repeated）：Ctrl LPS LPS+Mas-3μM**


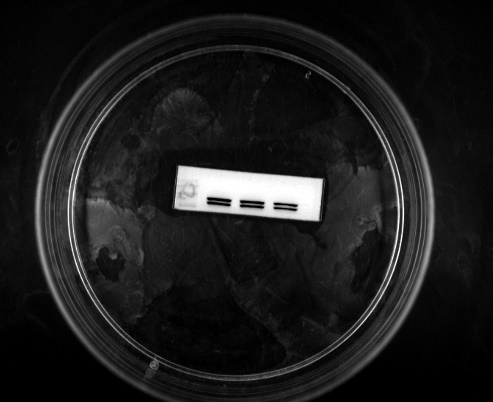




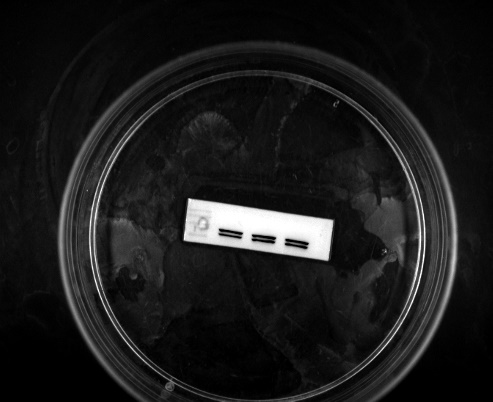




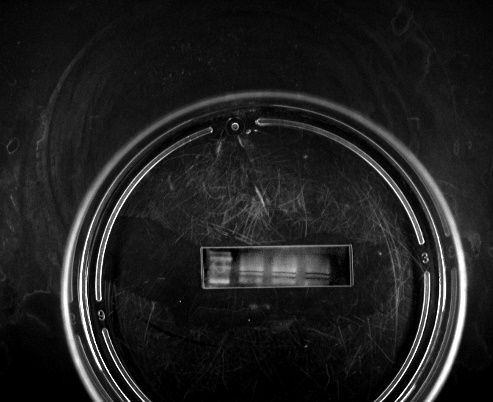




**Fig.8F**

**bax**

**（Used）：Ctrl LPS LPS+Mas-3μm**


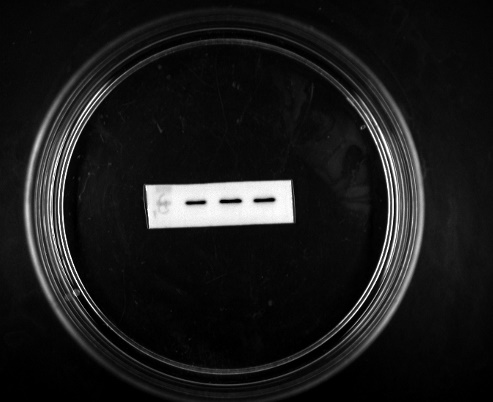




**（Repeated）：Ctrl LPS LPS+Mas-3μM**


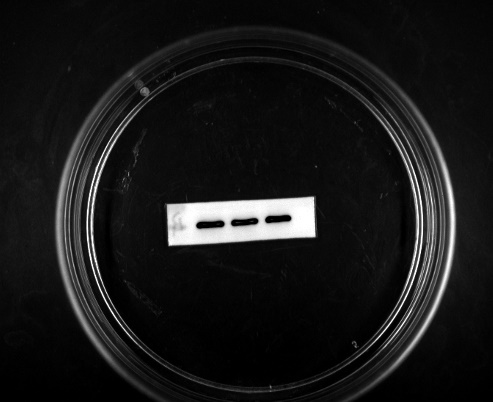




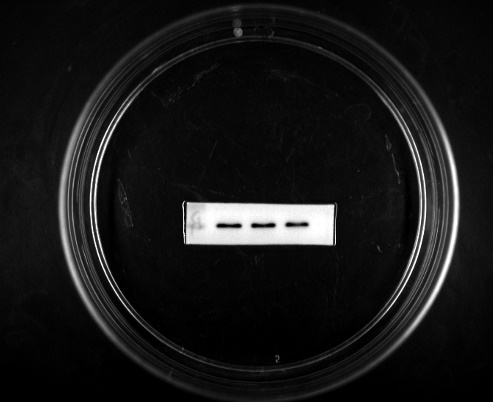




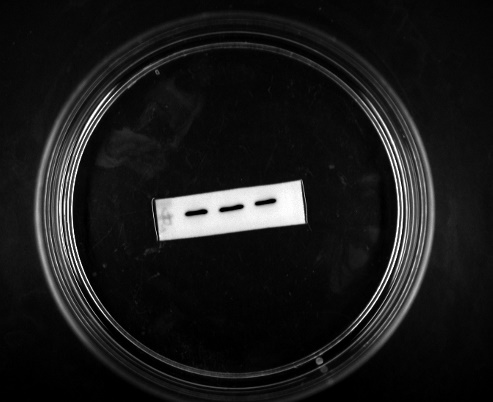




**Cyto-c**

**（Used）：Ctrl LPS LPS+Mas-3μM**


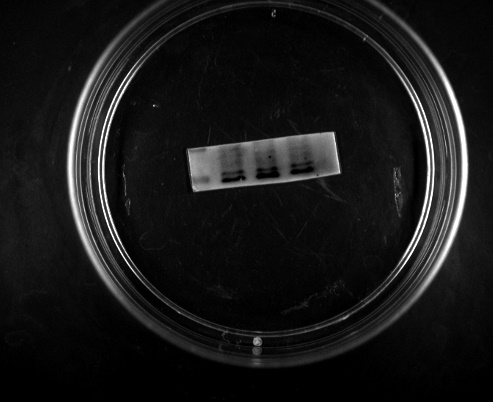




**（Repeated）：Ctrl LPS LPS+Mas-3μM**


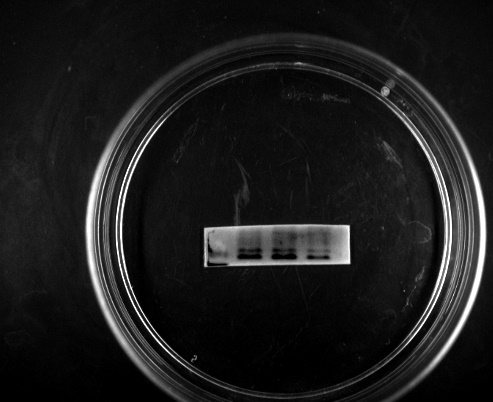




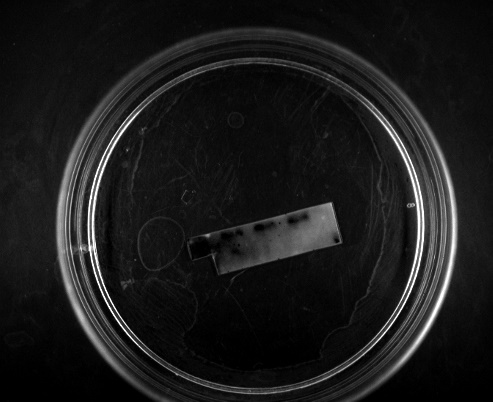




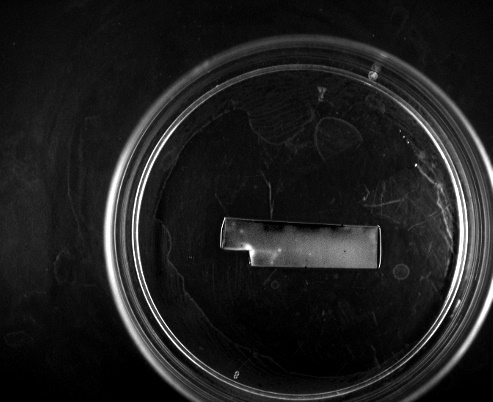




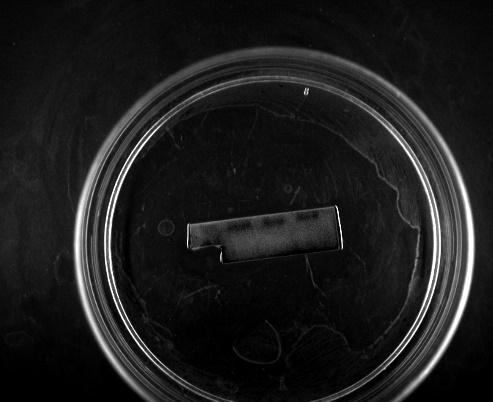




**Cleaved-caspase 3**

**（Used）：Ctrl LPS LPS+Mas-3μM**


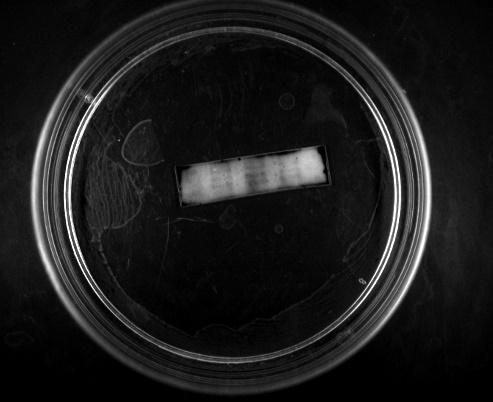




**（Repeated）：Ctrl LPS LPS+Mas-3μM**


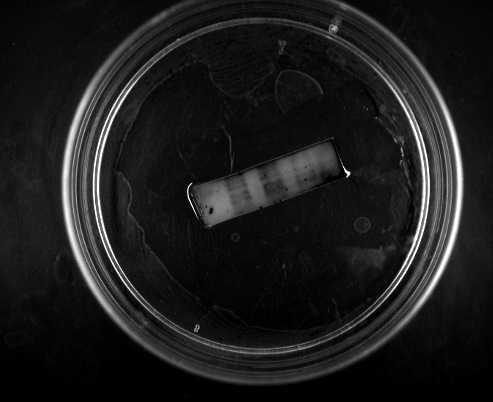




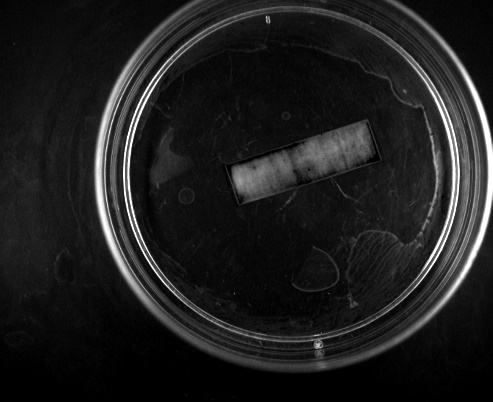

Supplement: Supplementary file 1 — Original Western Blots [file 41419_2023_6272_MOESM1_ESM.docx]
