# Supplementary figures and images for: Inhibition of PDGFRβ alleviates endothelial cell apoptotic injury caused by DRP-1 overexpression and mitochondria fusion failure after mitophagy
Source: Cell Death Dis. 2023 Nov 18;14(11):756. doi: 10.1038/s41419-023-06272-3 (PMC10657461; doi:10.1038/s41419-023-06272-3)

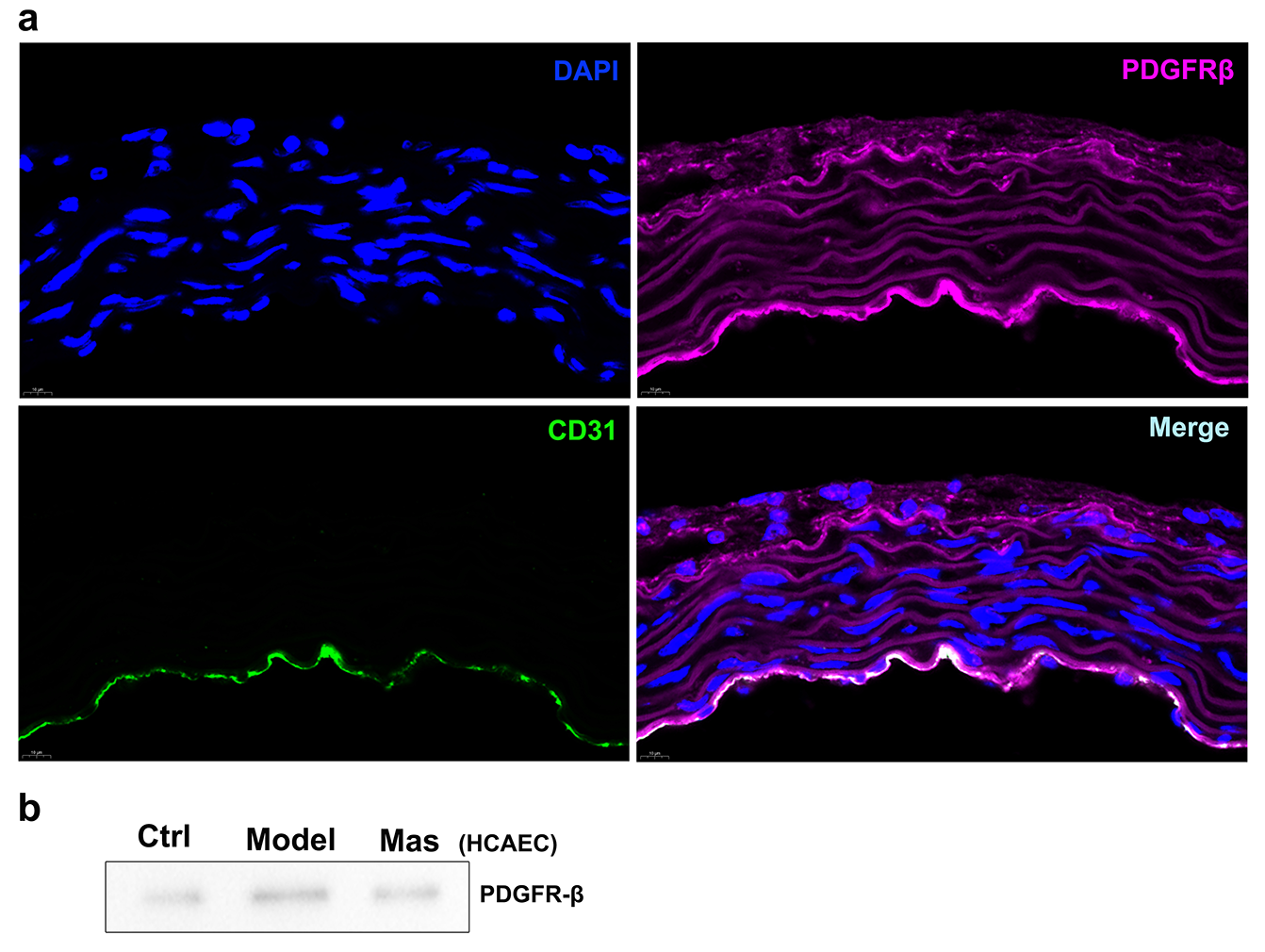

Supplement: Supplementary file 2 — Fig.S1 [file 41419_2023_6272_MOESM2_ESM.tif]
