# Supplementary material for: Inhibition of PDGFRβ alleviates endothelial cell apoptotic injury caused by DRP-1 overexpression and mitochondria fusion failure after mitophagy
Source: Cell Death Dis. 2023 Nov 18;14(11):756. doi: 10.1038/s41419-023-06272-3 (PMC10657461; doi:10.1038/s41419-023-06272-3)
Supplement: Supplementary file 3 — Figure S1 legend [file 41419_2023_6272_MOESM3_ESM.docx]

Fig.S1 **Hyperexpression of PDGFRβ in aortic endothelium of KD murine model and HCAEC.** **(A)** Representative immunofluorescence images for detecting PDGFRβ (magenta) expressions within aorta endothelium of KD murine model, CD31 (Green), DAPI (blue) (n=3). Scale bar: 10 μm. **(B)** Representative western blot analysis to determine the proteins expression of PDGFRβ in HCAEC cells.
